# Head movement and its relation to hearing

**DOI:** 10.3389/fpsyg.2023.1183303

**Published:** 2023-06-28

**Authors:** Nathan C. Higgins, Daniel A. Pupo, Erol J. Ozmeral, David A. Eddins

**Affiliations:** ^1^Department of Communication Sciences and Disorders, University of South Florida, Tampa, FL, United States; ^2^School of Aging Studies, University of South Florida, Tampa, FL, United States

**Keywords:** head movement, head-kinematics, listening intention, hearing aids, auditory spatial processing, augmentative and alternative communications systems

## Abstract

Head position at any point in time plays a fundamental role in shaping the auditory information that reaches a listener, information that continuously changes as the head moves and reorients to different listening situations. The connection between hearing science and the kinesthetics of head movement has gained interest due to technological advances that have increased the feasibility of providing behavioral and biological feedback to assistive listening devices that can interpret movement patterns that reflect listening intent. Increasing evidence also shows that the negative impact of hearing deficits on mobility, gait, and balance may be mitigated by prosthetic hearing device intervention. Better understanding of the relationships between head movement, full body kinetics, and hearing health, should lead to improved signal processing strategies across a range of assistive and augmented hearing devices. The purpose of this review is to introduce the wider hearing community to the kinesiology of head movement and to place it in the context of hearing and communication with the goal of expanding the field of ecologically-specific listener behavior.

## Introduction

Hearing aids and other assistive listening devices are on the cusp of major innovative leaps due to technical advances in machine learning, power sources, microchip miniaturization, increased capacity for online signal processing, and translational advances in the fields of auditory and cognitive neuroscience. Prosthetic hearing devices, are also uniquely positioned to capture key kinesthetic information due to their location on the head, providing great potential for capturing user intention based on head-movement as a listener interacts with the acoustic environment. Current hearing aid technologies typically rely on acoustic cues alone to determine which sound-features to amplify and which to suppress or modify (Ricketts et al., 2017). This approach, however, necessarily relies on many assumptions, leading to an inherent disconnect between device output and the intention of the user. Sensors that capture non-auditory cues such as head movements have the potential to provide a direct connection between the hearing device and aspects of listener-intention to automatically supplement device-processing and ensure a more productive listening experience.

In recent years, more attention has been paid toward individual variability in auditory perceptual abilities with the expectation that tailored approaches resulting from “precision audiology” will improve hearing health outcomes (Sanchez-Lopez et al., 2020). From the clinician’s perspective, there is still much unknown regarding the interaction of individual differences, other than what can be easily gleaned from basic audiological assessments. Due to the costs associated with additional tests in the clinic, there is a greater appetite for applications of “smart” technology that can learn functional characteristics of the individual wearer and adapt accordingly (Skoglund et al., 2022). For example, with knowledge of the behavioral patterns of an individual during active auditory and non-auditory activities, devices have the potential to anticipate whether a listener is engaged and participating in a conversation, and then adjust appropriately to the dynamics of head-movements and turn-taking patterns.

With the inclusion of inertial sensors (e.g., accelerometers or gyroscopes) in modern premium hearing aids, the devices are now capable of monitoring not just the acoustic environment but also the physical interaction of the listener with that environment (Fabry and Bhowmik, 2021; Rahme et al., 2021). Importantly, this approach avoids the need for explicit input from the listener via an interface like a mobile phone or remote control. Currently however, very little is known about the connection between the kinesthetics of head movement and the communicative goals of the listener or how individual differences influence these types of behavioral movements. Finally, it is still to be determined the best methods for harnessing that information and translating to improve hearing quality.

Studies of body language usually fall within the scope of fields such as social psychology, while investigations of the mechanical nature of head and body movements are common in the fields of kinesiology, physical therapy, occupational therapy, and exercise science. In an effort to combine information learned from across these disciplines to advance hearing enhancement technology, we review the relevant literature in the hopes that providing a resource that touches on a wide range of relevant head movement and balance literature will provide context, and promote advancement and communication between research, clinical practice, and the hearing device industry. This review is organized into five main focal areas: (1) the fundamental physics of head movement, (2) head movement during active listening (3) head movement during communication, (4) head-body interactions, and (5) the prospects of device intervention to benefit hearing and balance outcomes.

Many methodologies have been used over the years to study head movement, some with remarkable ingenuity (e.g., Zangemeister et al., 1981). A full accounting of these techniques is outside the scope of this review, but it should be noted that in this report (as with all studies), results are best interpreted in the context of the measurement equipment. Until recently, few widely available measurement systems were capable of simultaneously capturing rotational movements specific to yaw, pitch, and roll. Many contemporary recording systems track head location and orientation with 6-degrees of freedom, which allow for appropriate estimation of head azimuth and movement-kinematics, but are less interpretable for roll and pitch. Rotation-specific results are reported whenever possible.

## Physical properties of head movement

### Dimensions of the adult human head

To understand the connection between head kinematics and acoustic cues used in hearing, it is important to begin with the physical properties of the human head. The human head weighs up to 5.5 kg (12 lb) and has a circumference of approximately 60 cm (24 in), with average weight and circumference depending on age, height, and sex (Ching, 2007; Nguyen et al., 2012). Head size impacts interaural time (ITD) and level differences (ILD), demonstrated by comparative studies across animal species (Heffner, 1997) with vastly different head sizes.

**Figure 1:**
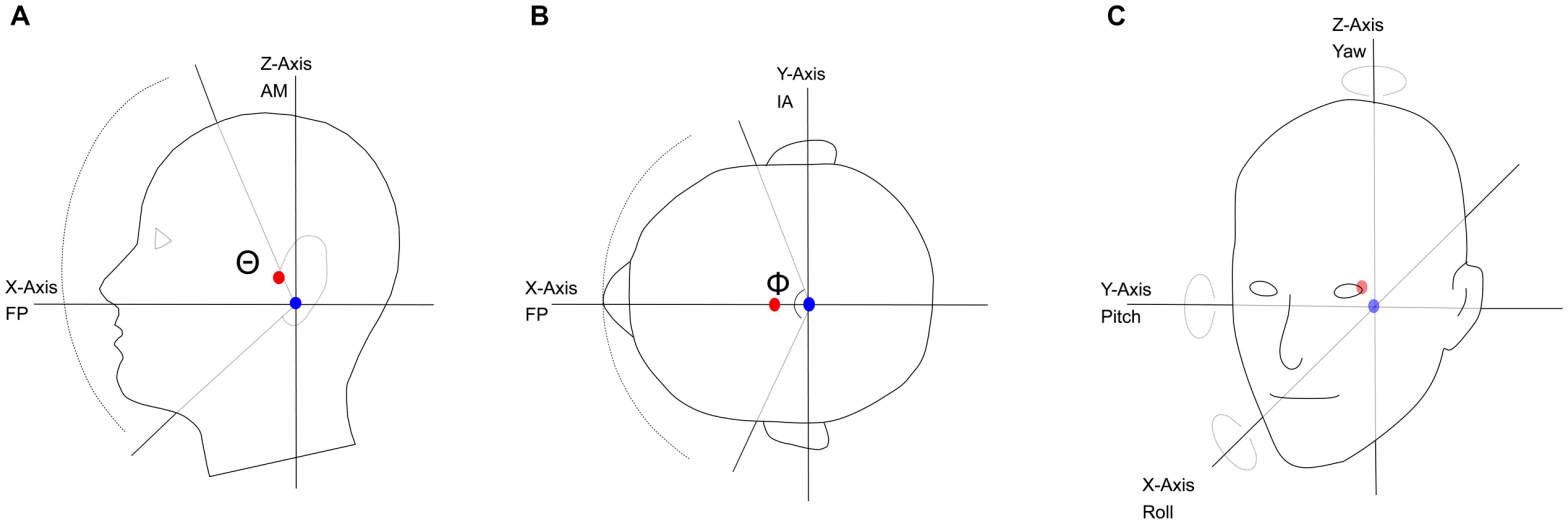
**(A)** The relative position of the center-of-mass and angle-of-rotation on the sagittal plane; nodding motion shown as a rotation around the Y-axis, with a maximum range of 170°(Θ). **(B)** The center-of-mass and angle-of-rotation on the axial plane; shaking motion shown as a rotation around the Z-axis, with a maximum range of motion of 160° (Φ). **(C)** Pitch, Yaw and Roll of the angle of rotation and the relative location of the center of mass. FP, Frankfort plane; AM, Auditory Meatus; IA, Interaural axis; blue circle, axis of rotation; red circle, center of mass.

Head size also dictates the location of the center of mass (Figure 1, red circle) of the head, which is the point where gravity acts to create a uniform distribution of weight. On average, the center of mass is located approximately 0.8 cm anterior to the auditory meatus, and 3 cm superior to the Frankfort plane on the sagittal plane (Figures 1A,B; Yoganandan et al., 2009). The Frankfort plane is the horizontal plane defined by the top of the left and right ear canals to the bottom border of the eye. The point corresponding to the center of mass is slightly off from the interaural- or pitch-axis of rotation (Figure 1, blue circle) that lies 0.38 cm posterior and 3 cm inferior to the auditory meatus (Moore et al., 2005). Differences in head size and shape affect the acoustic head shadow cast when sound sources are off-midline to one side of the head, and in theory, could impact the transition region between usable ITD and ILD cues (Cai et al., 2015).

### Axes of rotation: yaw, pitch, and roll

In addition to the standard axes of translation: x (rostral to caudal), y (left to right), and z (dorsal to ventral), there are three axes of rotation, each associated with one of the standard dimensions. The yaw axis rotates along the z-dimension (Figure 1C) characterized by a “head-shake” motion, a movement sometimes referred to as “axial-rotation.” The pitch axis rotates along the y-dimension (Figure 1C), characterized by a “head-nod” motion, a movement also referred to in the literature as “flexion” (head toward chest) and “extension” (head tilted back). Both the yaw and pitch axes of rotation are physiologically based on the atlanto-occipital and atlanto-axial joints (Bogduk and Mercer, 2000). Finally, the roll axis rotates along the x-dimension in a “side-to-side” motion, made possible by movement at the cervico-thoracic junction (Figure 1C; Bogduk and Mercer, 2000). The roll axis is also referred to as “lateral bending” in the kinesiology literature.

### Range of head motion

The maximal range of head motion along the **yaw axis** of rotation, is around ±80° with the majority of maximal ranges reported between ±60° and ±80° (Ferrario et al., 2002; Tommasi et al., 2009; Park et al., 2014; de Souza Melo, 2017). The yaw axis of rotation is more symmetric than the pitch axis, and range of motion is less impacted by aging compared to pitch (flexion or extension). Estimates of the maximal angle of rotation across a population on the yaw axis differs across studies, some reporting a full, 180° across the frontal hemifield compared to other estimates closer to 160° (Tommasi et al., 2009) or 175° (Dvorak et al., 1992).

Starting from rest at 0° along the **pitch axis** of rotation, the maximal distance while executing a flexion motion (moving chin to chest) is −90° and executing an extension motion (moving head to back) is +101° (Graf et al., 1995; Bogduk and Mercer, 2000). To achieve these angles, the atlanto-occipital joint average range of motion is approximately 15° (Bogduk and Mercer, 2000). For flexion motions, each of the cervical vertebrae can contribute up to 12° of additional motion, whereas for extensions, the vertebrae can contribute up to 15° of additional motion (Graf et al., 1995). The angle of motion increases with the vertebrae that are further down in the neck contributing more to the head orientation. This is due primarily to the combined ranges of motion for the atlanto-occipital and cervico-thoracic joints of the neck (Graf et al., 1995). Reports indicate that the maximal range of motion along the pitch axis decreases with age in non-pathological persons by about 10° from 20 to 50 years of age (Tommasi et al., 2009; Park et al., 2014).

From a starting point of 0° along the **roll axis** of rotation, the maximal range of lateral bending to the left or right is approximately ±35° to 40° (Ferrario et al., 2002; Inokuchi et al., 2015). Sex-related differences have been reported, with a significantly larger range of motion, close to 12° greater, reported in females than males (Ferrario et al., 2002).

### General kinematics

When the head orients to a target position, the **kinematics** of head movement conform to a predictive pattern of human movement known as Fitts’ Law: the time it takes to move to a target is a function of the distance to the target divided by the size of the target. This movement pattern can be described in two phases: (1) the *initial* movement, described by high velocity and imprecise change in position toward a target position; and (2) the *final* movement, which is slower, but more precise than the initial movement (Fitts, 1954; Chen et al., 2012). Demonstrating the efficacy of this model with regards to head movement (Hoffmann et al., 2017) observed that the time of movement along the yaw axis increased as the difficulty of target acquisition increased (via manipulation of target size), showing that head movements of 60° required 550 ms (109°/s) for the easiest targets, and 900 ms (66.7°/s) in the most difficult targets.

In studies that explicitly asked participants to quickly rotate their heads in the yaw domain, movement trajectories were sigmoidal in shape, with a near-linear relationship between the amount of rotation and the peak-velocity, reaching speeds up to and beyond 200°/s for large turns (Zangemeister et al., 1981; Thurtell et al., 1999; Kunin et al., 2007). In experiments where participants localized auditory targets presented across a loudspeaker array peak-velocity was highly correlated with the magnitude of a yaw-movement (i.e., jump size), ranging from 50°/s for small 30° jumps up to 150°/s or greater for jumps exceeding 90° (Brimijoin et al., 2010; Chen et al., 2012; Whitmer et al., 2022).

### Modeling head movement

**Figure 2:**
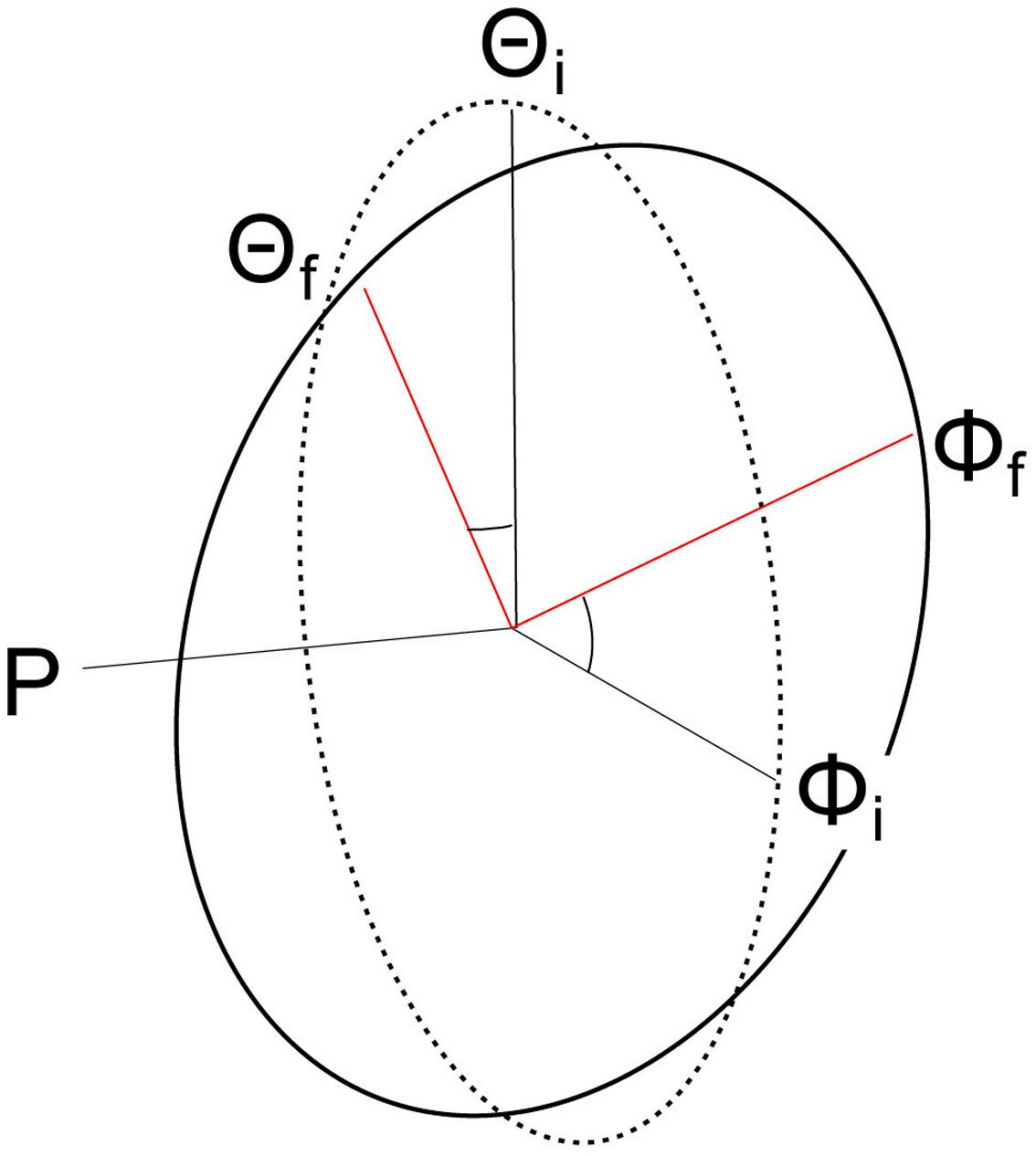
Diagram showing how head orientations move on Listing’s plane using a Fick Gimbal system. The Fick Gimbal system accounts for head movement that occurs along two planes (Θ = pitch, Φ = yaw) by considering all movement on the roll (rho) axis equal to zero. Listing’s plane is formed between the yaw and pitch axes and lies at the intersection for all the axes, or the axis of rotation. Movement from the initial (i) position to the final (f) position on the pitch and yaw axes occurs on Listing’s plane.

To accurately model head movement, the head can be considered a sphere (rigid body) rotating around a point such as the center of mass (Kunin et al., 2007); however, many models of head movement simplify the 3-dimensional (3D) sphere to either a 2-Dimensional (2D) or a 1-Dimensional system of equations. There are two primary tools for simplifying the 3D problem: Donder’s Law and the Fick Gimbal System (Figure 2). Donder’s Law states that starting from a frontal position, head orientation can only be obtained by a rotation matrix (quaternion) whose rotation is on a 2D axis. That is, the center of rotation must remain on the plane formed by the other two axes. When the head moves on the rotational yaw axis, for example, the model assumes that movement on the other axes (pitch and roll) are null or in the same direction (Figure 2). The Fick-Gimbal system describes 3D head movement by splitting the movement into its constituent components using a horizontal axis that directly intersects the vertical axis at the center of rotation as shown on Figure 2, known as Listing’s plane (Novelia, 2015). The center point of rotation is roughly midway between the ears and behind the eyes, as shown in Figure 1B (Moore et al., 2005; Kunin et al., 2007; Wijayasinghe and Ghosh, 2011). This axis is relevant for modeling turning or shaking head movements on the yaw axis.

## Head-movement during active listening

In nearly all naturally occurring environments there exist a variety of sound sources. Some of these sound sources are important while others can be safely ignored. Nevertheless, all sounds are transduced and refined to some degree at the auditory periphery, beyond which the central auditory processing system determines which sound sources require active attention (Gibson, 1966). In other words, all sounds are *heard*, but only some are *listened* to. To expressly measure active listening, research paradigms typically require an explicit participant-response to measure their perception, such as repeating back a word in noise or selecting between options in a discrimination task. A distinction can be drawn however, between measures of perception that are intuitive to put into words such as repeating back a sentence, vs. absolute measures or quality of a sound, such as of the speed of a sound-source or the distance or physical shape of an auditory object. Stroffregen and Pittenger (1995) make a compelling argument that for these types of experiments, the best approach to measure perception is with perception-action paradigms, where participants are asked to make an ecologically relevant action in response to the acoustic stimuli. Head movement is one of those ecologically relevant perceptually-driven actions, particularly for experimentation on the perception of sound movement, distance, and location.

### Sound localization

The ability to localize sound requires complex integration of interaural differences in time (ITD) and sound level (ILD) in combination with spectral cues specific to the individuals’ head-related transfer function (HRTF). Age-related hearing-loss followed by the introduction of hearing devices, fundamentally disrupts these spatial cues and the head-movement strategies the central auditory system relies upon for sound localization and auditory stream segregation (Van den Bogaert et al., 2006; Keidser et al., 2009; Kondo et al., 2012; Brown et al., 2015a).

### Sound localization: influence of subject response paradigms

In the vast majority of sound-booth or anechoic chamber sound-localization testing paradigms, participants begin with their head fixed at 0° azimuth and indicate their perception of sound location following stimulus presentation (Drennan et al., 2005). Participant responses are recorded in a variety of ways with varying degrees of head and body movement. Many paradigms use a pictured schematic of the sound field with labels marking spatial locations in the sound field, and ask participants to indicate with a verbal response or finger-point to the location on a touchscreen or paper in front of them (Lorenzi et al., 1999; Drennan et al., 2005; Keidser et al., 2006). This approach requires participants to generate an internal representation of the sound-space and translate that position to a recording device (Wightman and Jenison, 1995). Using an approach with an active-response and potentially more ecological relevance, other paradigms ask participants to physically point to the location with their hand (Brungart et al., 2017; Gessa et al., 2022), and others instruct listeners to point their heads in the direction of the sound source (Brimijoin et al., 2010; Brown et al., 2015a). Interestingly, the method of capturing participant responses appears to affect localization accuracy, with better performance observed when participants are free to move their head (Pollack and Rose, 1967; Grange and Culling, 2016b; Gessa et al., 2022), or receive linked visual-proprioceptive feedback such as an arm point to target or a laser-pointer (Otte et al., 2013; Ahrens et al., 2019). Further evidence demonstrates that a link between physical movement to the location of the sound facilitates adaptation to altered spatial cues (Valzolgher et al., 2020, 2022), supporting the hypothesis that perception-action paradigms are most relevant to this type of experimentation (Stroffregen and Pittenger, 1995).

### Sound localization: individuals with normal hearing vs. hearing loss

When instructed to orient their head to a sound source, similar patterns of movement were observed for both normal-hearing listeners and those with hearing-loss, but with different degrees of localization accuracy. Brungart et al. (2017) measured localization accuracy (arm point to sound source) and compared it to the total amount of listener head movement while localizing sounds in the free field. They found that during longer duration stimuli, 4,000 vs. 250 ms, participants had more time to make exploratory head movements, concluding that more total head movement led to improved localization accuracy. This result was observed in normal-hearing listeners who improved from 8° of azimuthal error to close to 0° of error. In parallel, the longer time duration for exploratory head movement time also led to a large accuracy improvement for the hearing-loss group, with decrease in azimuthal error from 35° to approximately 8°. This marked improvement indicates that individuals with hearing-loss maintain an ability to extract dynamic binaural cues accessed via head movement given sufficient time. Though Brungart et al. (2017) showed no significant head movement differences between the normal-hearing and hearing-loss groups, results from Brimijoin et al. (2010) showed greater complexity in rotational movements for the hearing-loss group, with greater choppiness in movement, and more changes in direction, quantified by a positive correlation between polynomial degree for best fit and the degree of hearing-loss.

### Sound localization: head movement strategies

In listening environments with a target-talker and masker-talker at a different azimuth, studies show that rather than pointing the head directly at the target-talker, the most beneficial head orientation is midway between the target and masker (Kock, 1950; Blauert, 2001; Jelfs et al., 2011). For large target-masker separations (≥90°), the greatest benefit was observed approximately 30° to 60° relative to the target (on the side of the masker), as this orientation maximizes binaural disparities and pinna cues (Jelfs et al., 2011; Grange and Culling, 2016b). Across listeners, high variability has been observed in the amount of head movement (or lack thereof) listeners will exhibit. Grange and Culling (2016b) showed in a spatial release from masking experiment with free-head movement, that while some individuals oriented directly to an optimal angle, others did not move at all, or appeared to move their head randomly, with movements greater than 10° on just 56% of trials. Similarly, in an experiment using an audio-visual virtual environment that simulated a multi-talker conversation, Hendrikse et al. (2018) observed negligible head movement when the visual component of the participant conversation was not presented.

One general conclusion from surveying the head movement literature, is that in experimental settings, unprompted listener head movement is heavily influenced by the context of the experiment and the directions provided by the experimenter. When listeners were explicitly instructed to explore their listening environment or if a visual representation of the talkers’ face was presented, head movement increased and performance on the speech in noise task significantly improved (Grange et al., 2018). Grange and Culling (2016a) showed that cochlear implant users obtained a significant benefit with a 30° head orientation relative to target with the masker at 90° or 180°, quantified as a 5 dB head orientation benefit for the young normal-hearing group, and 1.5 to 4 dB benefit for the bilateral cochlear implant and unilateral cochlear implant groups. In a simulation of real-life listening environments, the same group (Grange et al., 2018) showed a benefit of head orientation of 1.2 to 3.2 dB, a number influenced by variables related to reverberation and the number of distractor sources (Grange and Culling, 2016a). The investigators speculated that a 30° angle maximizes the head orientation benefit, while simultaneously maintaining the ability to access lip-reading benefits, and satisfies the social need to maintain gaze connection with the talker. The one exception to the lack of spontaneous, unprompted head movement in these studies, is from Brimijoin et al. (2012), who demonstrated that listeners with asymmetric hearing-loss will naturally turn their “better” hearing ear approximately 60° off-center from the target source.

### Spatial awareness of distance

In many listening environments, the presence of inanimate objects such as walls and objects create reflections and echoes. Listeners rely on these cues to create an internal acoustic-representation of their environment and configuration of objects relative to their physical location (Wightman and Jenison, 1995; Zahorik, 2002; Kolarik et al., 2016; Gandemer et al., 2017). Outside of a static environment, ITD and ILD cues reaching the two ears are ever changing due to movement of sound sources, acoustic reverberations, and head movements, referred to as dynamic ITD, ILD, and monaural spectral cues (Carlile and Leung, 2016; Stecker, 2018; McLachlan et al., 2023). One way the auditory system compensates for the oftentimes conflicting spatial information reaching the two ears is by weighting temporal information to emphasize the initial parts of sound and de-emphasize later parts of a sound (Wallach et al., 1949; Franssen, 1960; Brown et al., 2015b; Stecker and Moore, 2018), and by making small head movements to increase dynamic auditory cues and reduce confusions (Pollack and Rose, 1967; Gessa et al., 2022).

When asked to judge the distance of a sound, a dichotomy in perception is observed as near-sounds tend to be overestimated and far-sounds underestimated, with a transition point related to room characteristics (Zahorik and Wightman, 2001; Anderson and Zahorik, 2014; Parseihian et al., 2014). The cues used to estimate the distance of a sound are based on the ratio of direct energy to reverberant energy (DRR) and sound level cues, such that the DRR provides an absolute cue whereas the sound level provides a comparative cue useful for detecting changes in distance (Mershon and King, 1975; Bronkhorst and Houtgast, 1999; Kopčo and Shinn-Cunningham, 2011; Kolarik et al., 2016). Though human listeners are relatively poor at estimating the distance of auditory stimuli compared to visual stimuli (Anderson and Zahorik, 2014), there are a number of studies demonstrating high proficiency by listeners to use acoustic reverberations to gauge movement of the listening environment. Stoffregen et al. (2009), for example, used an experimental setup that featured a four-walled enclosure capable of moving back and forth in space while participants remained stationary. Participants were asked to sway, or move their head, in synchrony with the oscillation of the room while blindfolded and forced to rely solely on acoustic information to judge movement of the surrounding environment. Various conditions had loud-speakers mounted at the corners of the enclosure or placed on the non-moving floor of the enclosure. In both scenarios participants were able to match their head movements to oscillations of the enclosure-walls with high accuracy (Stoffregen et al., 2009).

In a study of distance perception that investigated the effects of training with various reaching strategies, and touches on the discussion above concerning subject-response paradigms, Hüg et al. (2022) demonstrated the power of integrating active physical movement with the perception of auditory space. Blindfolded participants tested in a pre-test, training, post-test paradigm, were presented with an auditory source and asked to reach their arm to the source-location on the table in front of them, or not to reach if the object was perceived to be outside of arm-range. Participants allowed to freely move their arm to explore the environment and find the auditory object during the training period outperformed the control group (that received extra practice) and the guided group (experimenters actively moved the participants’ arm to the source). All participants prior to training exhibited the overestimation of nearby sound-sources and underestimation of farther sound-sources described above, the group allowed to freely explore the environment came closest to having that bias extinguished in the post-test (Hüg et al., 2022).

### Echolocation

Echolocation, though typically associated with blind individuals and extreme examples in the animal kingdom, is a localization ability well documented in sighted, relatively untrained human listeners as well (Supa et al., 1944; Cotzin and Dallenbach, 1950; Stroffregen and Pittenger, 1995). In its simplest description, echolocation is a process whereby a sound is emitted and the resulting sound field is assessed, a feature of the environment changes, such as taking a step forward, and the process is repeated. Echolocation then is the interpretation of the comparison of the relative differences between the sound fields before and after the step forward, not much different than a carpenter knocking on one section of a wall and then another section to locate a wall-stud (Stroffregen and Pittenger, 1995). Described at the level of the two ears, this process can be considered in three stages: the initial self-generated sound emission, the overlap of the emission and echo, and then the echo only (Papadopoulos et al., 2011; Kolarik et al., 2014).

Performance of echolocators improves with concurrent movement of the head and body. Early research showed that blind as well as sighted (blindfolded) individuals were both able to walk down a hallway and accurately stop before encountering a flat reflective surface (Supa et al., 1944; Cotzin and Dallenbach, 1950). Rosenblum et al. (2000) had sighted blindfolded participants echolocate a wall set at varying distances while participants either stood still and emitted a self-generated emission of their choice or were allowed to move while emitting the self-generated emission. Results showed that judgments made while moving were closer to the target distance than stationary judgments (Rosenblum et al., 2000). When asked to identify 2-dimensional shapes, blind-expert echolocators were significantly better when they were free to move their head than when their head was fixed in position (Milne et al., 2014). In the Milne et al. (2014) study, specific head movements were characterized as anecdotal speculation that “echo saccades”, like eye saccades, provide additional “sound” snapshots to aid perception. In a follow-up study by Thaler et al. (2022) that tested blind echolocators’ ability to discriminate the position of a 7.5 mm wooden disk, results showed better localization for intermediate-lateral positions (45°) compared to midline (0°) or extreme lateral (90°) positions (Thaler et al., 2022). Further analysis of the binaural level and timing information reaching the two ears, revealed that the greatest rate of change in binaural intensity and timing differences as a function of azimuthal angle occurred for objects located at 35° to 55° relative to 0° midline. Even though the highest binaural cue resolution occurs at the midline, the point in azimuth where two side-by-side locations have the greatest difference in naturally occurring binaural cues is at that intermediate-lateral position (35° to 55°). Taken together, the findings of Milne et al. (2014) and Thaler et al. (2022) strongly suggest that given freedom of head movement, the echolocators used head position in the yaw domain to maximize the binaural information contrasted between successive repetitions of self-emissions.

Additional factors that influence echolocation include distance to and orientation of reflective objects (Rosenblum et al., 2000; Teng and Whitney, 2011), the frequency and duration of sound emission (Papadopoulos et al., 2011; Rowan et al., 2013), and experience as an echolocator (Teng and Whitney, 2011; Kolarik et al., 2014). Briefly, evidence shows that echolocators are most accurate (1) at relatively short distances between listener and reflective surface (less than 1 m), (2) when the emitted sounds have high frequency content, (3) when emitted sounds proceed for longer durations (performance improves as broadband stimulus duration increases from 10 to 1,000 ms), and (4) when the acoustic environment has high reverberation (Stroffregen and Pittenger, 1995; Rosenblum et al., 2000; Rojas et al., 2009; Schenkman and Nilsson, 2010, 2011; Papadopoulos et al., 2011; Rowan et al., 2013; Schörnich et al., 2013). It must be noted that strong variability is observed across individuals, particularly for very experienced echolocators. For a comprehensive breakdown of the factors affecting human echolocator performance, see reviews by Kolarik et al. (2014) and Thaler and Goodale (2016).

### Sound localization: temporal measures

In a complex acoustic scene, detection of and orientation to novel or relevant sound sources often requires multiple systems working in concert, including peripheral auditory processing (e.g., spectro-temporal resolution), central auditory processing (e.g., attentional capture), and fine-motor processing (e.g., head and neck movement). The timing of behavioral responses (i.e., reaction time) to new sounds therefore, can shed light on normally functioning or impaired functioning of these systems, and changes to the temporal course of behavior as a result of intervention may provide evidence of successful treatment options.

Numerous studies have shown that hearing deficits lead to slower and less accurate sound localization, as measured by head movements to source locations and ability to identify the speaker (Brimijoin et al., 2010, 2012; Archer-Boyd et al., 2015, 2018; Brungart et al., 2017). For example, the initial latency – the time it takes to initiate movement – is longer in unaided hearing-loss listeners relative to normal-hearing listeners and it takes listeners with hearing-loss longer to arrive at a target location (i.e., fixation latency) than normal-hearing listeners (Brimijoin et al., 2010). This latter observation may be confounded by the age of the hearing-loss group vs. the normal-hearing group. When searching for a sound source location, listeners with hearing-loss tend to have more reversals and “inconsistent variations” in their movement, which could be due to poorer cognitive or motor function as opposed to hearing deficits (Brimijoin et al., 2010). Considering age-related hearing loss very often manifests at higher frequencies, there also appears to be a frequency dependent relationship between the time to initiate head movement and head movement accuracy. Whitmer et al. (2022) demonstrated that listeners with minimal hearing loss performed more accurately and made faster initial movements towards acoustic targets that were low-pass filtered at 10 kHz compared to 5 kHz.

## Head movement during communication

Head movements in communication settings comprise a variety of head movement patterns with a correspondingly wide range of culturally specific meaning and interpretation. For most western cultures, the most common communicative head movements are “nods” and “shaking,” both of which involve oscillations along the rotational axes (Kousidis et al., 2013; Wagner et al., 2014; Hall et al., 2019), though the meaning of these movements can vary by culture (Andonova and Taylor, 2012; Wagner et al., 2014).

“Turn” and “tilt” head movements are also described with similar frequency of occurrence in communication environments, with each motion leading to a different pattern of movement around the pitch, yaw, and roll axes of rotations. Together, all of these movements affect the nature of the sound received by the ear in a manner that depends on the axis upon which the head is rotating and the frequency of the head movement (Altorfer et al., 2000). For example, a turn-and-tilt movement to point a better-hearing ear toward a sound source that increases binaural disparity, involves integrated rotation in the yaw, roll, and pitch dimensions (Brimijoin et al., 2012).

Each of these movements has the potential to convey meaning on their own or in combination with vocalization (Munhall et al., 2004). Over the time course of dyadic conversation for example, different head movements are observed for the listener and the talker. When in the speaking role, a person is engaged in behavior that is meant to convey a message. When in the listening role, a person is engaged in behavior suited toward absorbing information and understanding meaning (Boker et al., 2009; Wagner et al., 2014). The kinematics of the head reflect these roles. In the speaking role, movements are used that depend on the meaning they want to convey (Munhall et al., 2004). For example, rapid head movements may be used to take control of the conversation, such as when interrupting another speaker (Hadar et al., 1983) or making positive assertions (Bousmalis et al., 2013). In the listening role individuals adjust their heads for improved auditory cues, to convey understanding prior to the speaker completing a sentence, and when presented with more difficult listening environments, such as louder background sound, listeners will move their head closer to the talker (Hadley et al., 2019). Also documented, is an element of head movement mimicry, where listeners will reflect back their partners’ head movements in both the horizontal and vertical planes (Boker et al., 2009; Hale et al., 2020).

Head movement between conversational partners has also been shown to provide nuance to observers. For example, observers were able to identify whether interactions were between friends or strangers with a higher success rate when presented head and face movements compared to body movement, indicating that head movement communicates familiarity between talkers (Latif et al., 2014). Observers were also able to accurately identify “real dyadic interactions” compared to pseudo (unconnected) interactions (Bernieri, 1988). Head movements in this context were characterized by intensity, the rate of movement, magnitude of displacement, range, and velocity, and expression of strong emotion has been shown to increase the intensity of head movements (Wagner et al., 2014). This emotion and intensity can be expressed further through singing (Livingstone and Palmer, 2016) or attempted deception (Duran and Fusaroli, 2017). For example, when deceivers disagreed with the target of deception, they anticipated the coming responses, provoking an immediate response to their conversational partners head movements and an increased amount of coordinated head movements and mimicked movements on the part of the listener (Duran and Fusaroli, 2017). When there is more agreement between conversation partners, more head movement and greater speech rate coordination are observed (Boker et al., 2009; Duran and Fusaroli, 2017).

### Yaw: side-to-side head shake

While listening to speech stimuli, individuals generally orient their head in azimuth to the direction of the target, but often with a systematic offset between head angle and location of the talker. Movements in the yaw domain have a linear relationship to target angle or talker, quantified by a slope of approximately 0.6, representing a consistent “undershoot” of head turns (Stiefelhagen and Zhu, 2002; Brimijoin et al., 2010). In multi-talker conversations, this undershoot corresponded to a range of head angles 10° to 15° short of the target talker while eye gaze compensated for the difference (Hendrikse et al., 2018; Lu et al., 2021; Lu and Brimijoin, 2022).

Movement along the yaw axis is prominently involved in active listening strategies and non-verbal communication. There are two very similar types of movements, one described as an orienting, non-cyclical head-turn along the yaw-axis of rotation, with slight movement on the roll axis (Kunin et al., 2007). The other is a head-shake, with properties similar to a head turn but with a repeated, periodic rotation of the head, typically observed ±30° about the midline (Hadar et al., 1983; Moore et al., 2005; Kunin et al., 2007). Both movements lead to simultaneous changes in the position of the ears with respect to the point of rotation and sound source (Figure 1C). Both of these movements result in the potential for increased localization capacity due to changing ITDs, ILDs, and spectral notch differences associated with the head-related transfer function (Brungart et al., 2017), and a substantial reduction in front-back confusions (Yost et al., 2020; McLachlan et al., 2023). Thus, a head turn provides a listener-initiated increase in available spatial information, and greater sensitivity for distinguishing a target auditory source from background noise, an advantage that can be quantified with a spatial release from masking measure, as defined in the hearing literature (Cherry, 1953).

Head shaking has a similar potential for increasing spatial information and serves as a means of non-verbal communication. The nuances that carry specific meaning in head movements can be difficult to study due to challenges in systematically defining and annotating specific behaviors across multiple observers (Kousidis et al., 2013). Available evidence indicates that varying levels of intensity, velocity, and range are the key features that carry communication intent, transmitting signals such as feedback from a listener to a talker, indicating an interest in contributing to a conversation (turn-taking), and providing emphasis or subtlety to speech delivery (Hadar et al., 1983; Wagner et al., 2014). While rapid changes in pitch, or head nodding, has been implicated as a conveyance of both positive and negative connotations, head shaking has been hypothesized to be a complex unit of expression, but almost always conveys negativity (Kendon, 2002). This negativity can range from expressions of uncertainty or confusion to impatience to vehement opposition with a variety of expressions in between (Kendon, 2002; Heylen, 2008). A comprehensive characterization of the underlying kinematics for all of these different communications has not been compiled, but reports of head shaking during normal conversation indicate an oscillation range from 0.2 to 7 Hz, and this range has been subdivided into slow movements for frequencies between 0.2 and 1.8 Hz, ordinary movements between 1.9 and 3.6 Hz, and rapid movements with frequencies between 3.7 and 7.0 Hz (Hadar et al., 1983).

### Pitch: up-down nodding

Movement along the pitch axis of rotation can take the form of a single downward (flexion) or upward motion (extension) made in the context of several activities, but in the field of active attention is naturally suited to surveying the external environment along the vertical dimension. Periodic pitch movements are often characterized as a “nod” in the context of communication. This motion is defined as repeated sinusoidal oscillations along the pitch axis combined with little movement along yaw and roll axes (Moore et al., 2005; Kunin et al., 2007). Nods naturally occur over a range of −3° (flexion) and +15° (extension) on the pitch axis of rotation during active listening (Hendrikse et al., 2019). This oscillation, while maintaining the external auditory meatus (ear canal) in space on the left and right sides of the head, does lead to some incremental movement in a vertical direction (z-axis of translation) with flexion and extension of the neck over a range of approximately ±10 mm (Moore et al., 2005). The result of this movement is that during a nod, the point of rotation minimally effect the position of the ear canals (Moore et al., 2005), while changing the orientation of the pinna, modulating the spectral information available to the listener (Hirahara et al., 2010), and potentially improving speech perception (Grange and Culling, 2016b; David et al., 2017; McLachlan et al., 2023).

Of the synchronized behaviors and mimicry used to facilitate social interaction, head nodding acts as one of the primary means for conversational back-channeling, a term used to describe activity that confirms attention and encourages the talker to continue, and confirms mutual interest (Kendon, 1970; Lakin et al., 2003; Hale et al., 2020). In a study by Hale et al. (2020), analysis of dyadic conversations showed that movements in the pitch dimension exhibit strong similarity between participants, quantified by a strong positive coherence of movement for frequencies between 0.2 and 1.1 Hz, with a 0.6 s lag for conversation organized as a leader and a follower. Conversely, coherent movement between the two participants was *not* observed at low frequencies for yaw and roll (Hale et al., 2020). At higher frequencies (2.6 to 6.5 Hz) a significant lack of coherence was observed, where listeners made many more “fast nods” than talkers, providing yet another avenue for non-verbal communication between conversational partners.

### Roll: left–right head tilt

Head tilts also are noncyclical and differ from nodding, shaking, and turning in that the rotations are on the roll axis (Kousidis et al., 2013; Wagner et al., 2014). In the context of non-verbal communication during conversation, the meaning of head tilts is harder to define and annotators often confuse them with turns (Kousidis et al., 2013), but have been proposed to indicate disbelief, uncertainty, and skepticism (Stone and Oh, 2008; Wagner et al., 2014).

## Head-body interaction

The intuitive connection between hearing, head stability, and balance is that auditory information allows the listener to navigate the environment, avoiding obstacles and hazards, and providing feedback about self-movement (Cornwell et al., 2020). From a biological perspective, the auditory periphery and vestibular system also share anatomical space, blood circulation, fluid-filled compartments, the eighth auditory nerve, and a number of co-occurring conditions such as Meniere’s disease, retro-cochlear lesion, and ototoxicity (Katz et al., 2002). The following sections further explore the relationship between head movement, hearing, and the maintenance of balance.

### Head stability as it relates to standing balance

The head contains most of the sensory organs associated with stability including the visual system, the auditory system, and the vestibular system, and head stabilization is vital to the maintenance of balance and gait orientation whether standing still or navigating the environment (Bogduk and Mercer, 2000; Peterka, 2018). Head stability is facilitated by maintaining standing balance through adjustments in the trunk or lower legs (e.g., ankles). Slight corrections in the trunk or lower body, which people do not generally notice, act to balance the head as well as the entire upper body (Baird and Van Emmerik, 2009) and can be measured by the amount of *postural sway*. When standing in place and turning, people can rotate their head to look over their shoulder about 60° or 70° on the axial (yaw) plane (Figure 1B), independent of trunk movement (Baird and Van Emmerik, 2009). When looking at targets that are outside a person’s field of view, people tended to comfortably move their heads either 60° or 70° on the yaw axis, and 40° or 55° on the pitch axis. The velocity of these movements ranged from 200 to 600°/s, with an average velocity of 271°/s and slower movements for the pitch axis than the yaw axis (Paquette et al., 2006). These results depend on the amount of restriction in motion a person has and the age of an individual, with older adults and people with more restricted movements showing less angular movements (Paquette et al., 2006; Baird and Van Emmerik, 2009).

During a loss of balance, an individual must correct their head orientation by making a corrective movement in the form of a step to regain balance, thus allowing the individual to reorient their posture and regain their balance. The ability of a person to regain balance depends, in part, on the time it takes to step and the velocity of the head (Diehl and Pidcoe, 2011; Kowalewski et al., 2018). Head velocity in translation (rather than rotation), during the transition from a step to a standing balance is also an important consideration in head stabilization. Peak velocity, the highest measured velocity within a specified span of time is useful in these studies for quantifying compensatory movements. In a study where balance was disrupted and individuals were forced to readjust, peak velocity ranged from 150°/s and 100°/s under normal conditions with latency to step initiation measured at approximately 0.30 s. Peak velocity and step latency were strongly correlated, with r values > 0.80: as peak velocity increased, step latency increased for both younger and older cohorts (Diehl and Pidcoe, 2011). In general, older adults also exhibited greater angular velocity than younger adults indicating greater stability compensation (Diehl and Pidcoe, 2011).

To discuss the relationship between standing balance and hearing, a brief background on theories of standing balance is helpful. Current understanding is that the postural stability needed for balance involves integration of multiple sensory (somatosensory, visual, auditory) and proprioceptive modalities that work in tandem with the motor system in a feedback and feedforward relationship. Experiments examining the relative contributions of different sensory systems indicate a weighted distribution that shifts as available sensory information changes (Dozza et al., 2007; Assländer and Peterka, 2014; Peterka, 2018). For example, when participants were are asked to stand still on a force plate while blindfolded (vision disrupted), the amount of sway increases compared to the not-blindfolded condition (Letz and Gerr, 1995; Maurer et al., 2000; Kanegaonkar et al., 2012; Zhong and Yost, 2013). Explained by theoretical models of compensation, in this scenario somatosensory, auditory, and proprioceptive information would be given greater weight and additional cognitive resources called on; with the final result that the person, while not falling or making a compensatory step, does exhibit greater postural sway (Dozza et al., 2007; Peterka, 2018).

Studies have also demonstrated the use of auditory cues in the environment to reduce sway (Zhong and Yost, 2013; Ross and Balasubramaniam, 2015; Stevens et al., 2016; Vitkovic et al., 2016; Gandemer et al., 2017). Easton et al. (1998) for example, had sighted (eyes closed) and blind participants place their feet in the tandem Romberg stance (heel-to-toe) and measured sway via force plate as well as head movement sensors. They found a significant reduction in sway for both groups when two loudspeakers placed at the level of the pinna presented a 500 Hz square wave compared to the no-sound condition. They also demonstrated large correlations (>0.75) in all experimental conditions, between sway measured with the force place and sway measured by head movement sensors (Easton et al., 1998). Dozza et al., 2007 showed reduction in sway by implementing an auditory biofeedback loop that used accelerometers to track postural sway and update sound delivery in real time (over headphones), with sounds that corresponded to the degree and direction of the body accelerations. In studies that used non-speech stationary sounds, the decrease in sway was relatively small, around 9–20% compared to no-sound (Easton et al., 1998; Zhong and Yost, 2013; Ross and Balasubramaniam, 2015), an amount that increased to 30% reduction with rotating sound (Gandemer et al., 2014). Deviterne et al. (2005) compared postural sway in elderly participants following physical rehabilitation for locomotor apparatus surgery and showed significantly greater sway reduction when 20 s stories were presented from a slowly rotating sound source (0.2 revolutions per second), compared to a slowly rotating pure tone.

It must be noted that in addition to studies that did not show an effect of auditory stimuli to reduce sway (Raper and Soames, 1991; Park et al., 2011; Seiwerth et al., 2020) a number of the experiments highlighted above had test conditions that did not demonstrate reduction in sway with auditory stimuli. Easton et al. (1998) for example, did not see a reduction when a single speaker was placed in front of the participant and presented the same 500 Hz square wave, and Deviterne et al. (2005) did not see reduced sway in the pure tone condition compared to the control condition with no sound. Taken all together however, the evidence indicates that maintenance of standing balance can include an auditory component depending on the availability and quality of visual and somatosensory input, but that the relative weight of auditory information is small (Dozza et al., 2007; Peterka, 2018). Furthermore, comparison of the studies that have shown an effect of auditory stimuli on standing balance with those that did not, it can be speculated that the usefulness of the auditory cues depends on their spectral complexity and reliability, the same sound features used to generate spatial awareness (Gibson, 1966; Wightman and Jenison, 1995).

As individuals age, sensory and proprioceptive systems begin to decline and their utility for providing feedback to the motor system is compromised. In order to maintain competency in daily life individuals compensate via reallocation of cognitive resources (Humes and Dubno, 2010; Schneider et al., 2010; Seidler et al., 2010), often measured with dual-task paradigms that require participants, for example, to perform an n-back memory task in difficult or easy listening environments while balance is perturbed (Bruce et al., 2019). In that experiment, as the auditory task became more difficult, standing balance was disrupted and greater amounts of sway were observed. Growing evidence indicates that auditory deficits can precipitate cognitive decline, further limiting the pool of compensatory cognitive resources available and increasing the chances of an adverse postural event such as a fall (Sibley et al., 2015; Agmon et al., 2017). For more on the connection between age-related hearing loss and cognitive decline, see Rönnberg (2003) and Rönnberg et al. (2022).

### Head stability as it relates to the gait cycle

During activities that involve full body movement such as gait, the head is used for balance. Normal gait in this context refers to the gait of non-pathological, healthy adults at an average walking speed. In steady state, or controlled balanced walking, a person requires coordinated control of the trunk movements and neck muscles to maintain the head’s center of mass (Cromwell, 2003; Cromwell et al., 2004;Keshner, 2004; Kavanagh et al., 2006). The head has to be balanced on top of the person’s trunk to walk in a stable manner, which is known as trunk-dependent movement (Keshner, 2004; Kavanagh et al., 2006). When trunk movement is restricted, balancing the head becomes more difficult and the head moves independently of the body. When trunk movement is unrestricted, the person can use the trunk to balance the head and control head movements (Keshner, 2004; Kavanagh et al., 2006).

**Figure 3:**
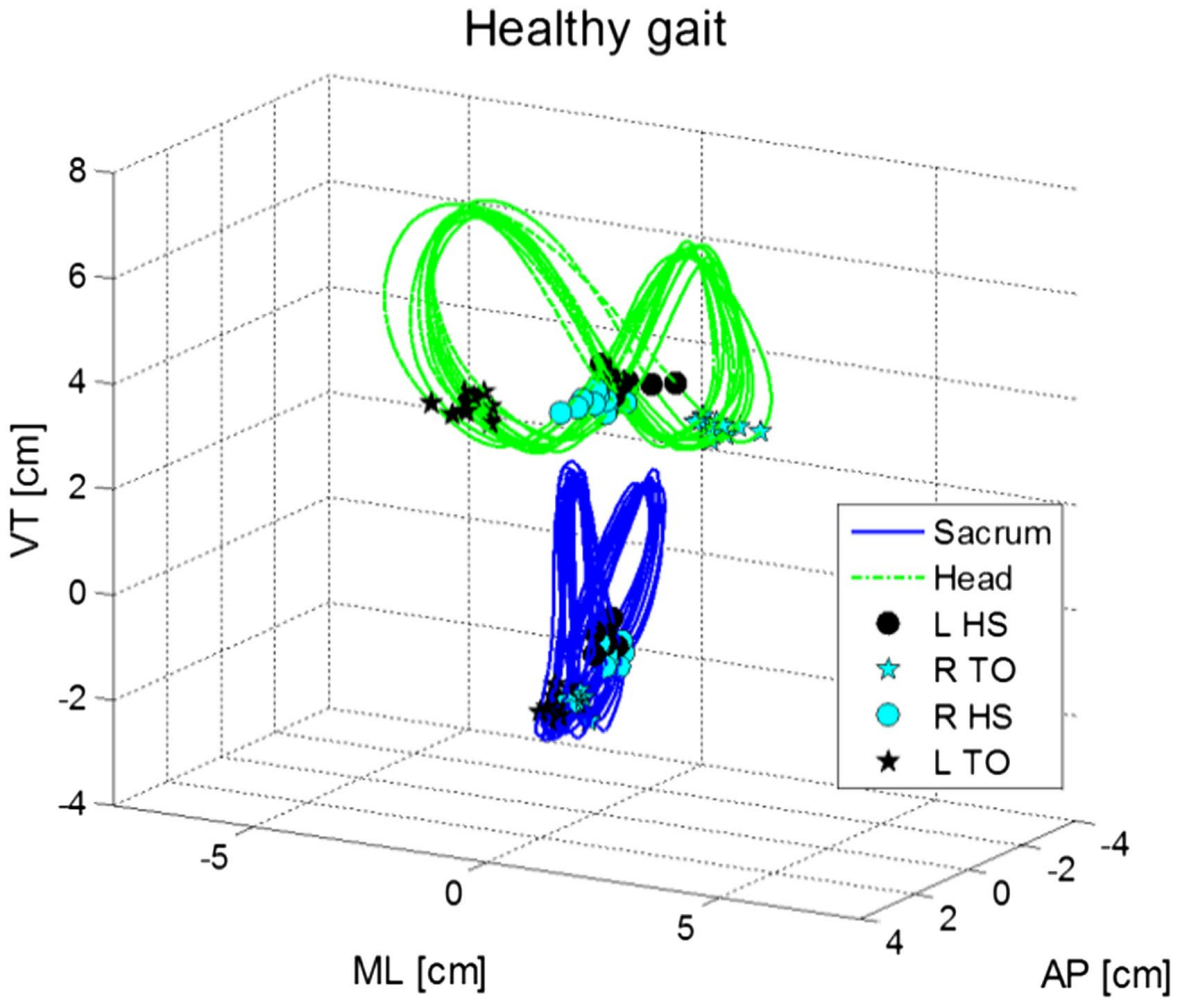
Shows movements in the vertical (VT), medio-lateral (ML) and anterior–posterior (AP) motions for the head (green) and pelvis (blue) in normal gait for a healthy young adult. Figure marks the points of left and right heel strikes (HS) and toe off [TO; from Brodie et al., 2014].

Additionally, during gait the head moves in repeated side-to-side and front-to-back oscillations (Figure 3). These head movements, which appear in an elliptical formation around the person’s center of mass, are synchronized with the gait cycle. Walking speed and step length are generally independent of head stability (Hirasaki et al., 1993; Brodie et al., 2014). Movement along the pitch dimension compensates for the translation of the head in the z-dimension (up-down) during the gait cycle, as head position goes up, the head tilts forward (anterior), whereas when head position goes down, the head tilts back (posterior). The range of pitch motion during this cycle has a range of approximately 10° (Hirasaki et al., 1993).

**Figure 4:**
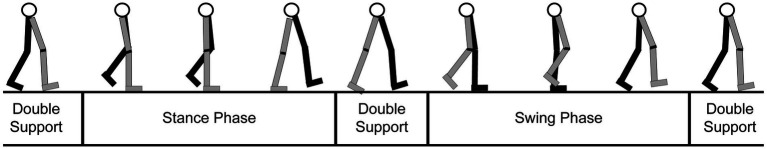
Diagram of the gait cycle tracing the movements of a leg through the stance, swing, and double support phases. Gray: right leg; Black: left leg (adapted from: https://clinicalgate.com/assessment-of-gait/).

The gait cycle is divided into different stages, based upon the location of the person’s feet and body. Each stage focuses on the path of motion of one leg since the other leg will mirror the movement (Stöckel et al., 2015). As illustrated in Figure 4, the stages are (1) the double support (both legs are on the ground), (2) the stance (one leg is planted on the ground and the other leg is in the swing phase), and (3) the swing (one leg is off the ground and “swinging” forward, while the other leg is planted).

During the gait cycle, there are two cycles of head acceleration corresponding to the movements of the left and right foot. These cycles of acceleration correspond to the moment the foot contacts the floor and the upward swing upon the foots’ release. Thus, head movement during the gait cycle can be divided into the contact phase of the foot and the foot release (Hirasaki et al., 1993; Brodie et al., 2014). The effect of hearing-loss at these two different points may be related to how sound is augmented by different accelerations (Campos et al., 2018). Of course, this also depends on the frequency of the head moving, which has been found to move from side to side in an ellipse at about 1.75 Hz for both younger and older healthy individuals. These results are similar to the frequencies reported by Hadar et al. (1983) as well as Brodie et al. (2014).

As a physical activity, walking requires coordinated integration of a number of different systems, and gait speed is a useful indicator of overall health quality (Studenski et al., 2011). Much of the literature linking the gait cycle and hearing have investigated the impact of hearing-loss on aspects of the gait cycle. In a statistically controlled study with a large cohort (*N* > 1,000), gait speed was significantly slower in individuals with hearing loss, characterized by an estimate that a 25 dB shift in hearing loss was equivalent to approximately 12 additional years of age (Li et al., 2013). Viljanen et al. (2009) in a cohort of 434 women, provide an age-adjusted estimate that hearing loss doubled the risk of having major walking difficulties compared to those without hearing loss. Directly connecting the auditory feedback provided by footsteps, in an age-adjusted sample of older adults, the amount of time spent in the double support stage of the gait cycle (Figure 4) was significantly higher in participants with elevated high- and low-frequency pure-tone thresholds (Szeto et al., 2021).

The presence of environmental acoustic stimuli has also been shown to impact the gait cycle. Cornwell et al. (2020) demonstrated that older, normal-hearing adults made significantly longer step lengths when wearing earplugs compared to no earplugs control condition, hypothesizing that lack of auditory feedback prompted participants to seek greater somatosensory feedback provided by foot-ground strikes. In a group of young normal hearing listeners, a reduction in angular velocity (measured near to the body’s center of gravity) was observed in a variety of walking tasks including eyes open, and tandem steps when continuous noise was presented compared to silence (Anton et al., 2021).

Irregular gait is a term that describes how a person walks during non-ideal circumstances such as incline walking and turning. Dampening effects of the trunk are commonly observed during both turning and incline walking. When turning, head rotation is greater in younger adults, who presumably are better able to stabilize their gait compared to older adults (Paquette et al., 2006; Baird and Van Emmerik, 2009; Kuo et al., 2014).

### Head movement during incline walking

**Figure 5:**
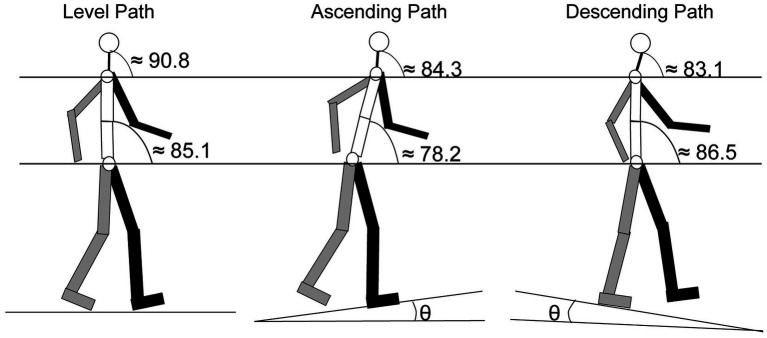
Segmented average angular orientation for the trunk and the head for level, ascending and descending paths. Gray: right arm and leg; Black: left arm and leg [ɵ = 8.5°; adapted from Cromwell, 2003].

One of the most common orientation changes that occurs when walking happens when encountering an incline. The change in the body’s orientation forces the head angle (Figure 5) to adjust in order to maintain balance, and modulates the orientation of the of the ears along the pitch axis (Figure 1) and sound cues presented to the ears are augmented (Hirahara et al., 2010). Cromwell (2003) compared head orientation while walking on a level plane to walking up or down an incline of 8.5°. Results showed that participants held their heads at an average position of 90.8° (±6.6°) when walking on a level plane. When walking on an incline, the average position decreased to 84.3° (±8.6°) and when walking on a decline, the average position declined to 83.1° (±9.8°). These changes in head position were significantly different from each other. The decrease in angle during inclined and declined was a result of changes in orientation of the neck and lower body (Figure 5). In the same study, Cromwell observed the amount of “excursion” the head made during strides. Defined as the average distance the head traveled between successive steps, excursion measures showed that during level-plane walking, the participants swayed their heads 5.6° (±1.6°) on the pitch axis, an angle that increased to 9.8° (±2.4°) for both the incline and decline paths (Cromwell, 2003).

### Head movement while turning and walking

Walking along a linear path is the most common form of locomotion, though there are times in which people are required to make turns. Movements involved in turning can range from pivoting over the hips, to slight alterations in body posture (Paquette et al., 2006), to making 180° turns mid stride (Kuo et al., 2014), and turning to look behind (Baird and Van Emmerik, 2009). Of particular interest is how head movements are affected when turning involves a change in gait pattern (Paquette et al., 2006; Kuo et al., 2014). Paquette et al. (2006) measured head movement during a goal directed task, where participants walked and then moved their heads toward targets. The average head turns were between 70° and 80° when walking, with the greatest head turns occurring for turns on the yaw axis (Paquette et al., 2006). Kuo et al. (2014) reported head movements for the phases of turning, notably that the swing phase (Figure 4), in which turning occurs, was characterized by less angular displacement in older adults, with turns of 23.65°, than younger adults, with head turns of 26.02°. From the perspective of audition, turning the head can allow more precise localization of specific objects (Grange and Culling, 2016b) and may impart a more complete awareness of the interactions between room acoustics and room characteristics (Yost et al., 2020). Thus, head movement while turning may produce important auditory sensations that facilitate the maintenance of balance throughout the turning and gait motions.

### Head and eye movement interaction

Finally, it is worth considering the eyes as a third independently moving structure apart from the head and body. Undoubtedly there is synergy between head and eye movements, in that simple unidirectional head movements generally correspond to the head and eyes moving in unison (Collins and Barnes, 1999; Land, 2004). Research has shown however, that these variables can be more dependent or less dependent based on the task and environment. During simple walking, for example, individuals tended to scan the environment with a range of approximately 10–30° in the horizontal plane (Franchak et al., 2021). During this activity, eye and head angle were mostly in agreement, but about 16% of the time the two angles had a different sign [e.g., head pointed left while eyes pointed right (Tomasi et al., 2016)]. During this activity, head movement contributed about 45% to gaze shifts (sum of eye and head rotation) that ranged from 10 to 60°. For gaze shifts greater than 60°, head movement contributions increased to about 60% or higher, while the eye movement contribution decreased (Franchak et al., 2021). As walking gets more complex due to increasingly difficult terrain, gaze shifted downwards in the pitch domain approximately 30°, of which head movement accounted for approximately 15° to 20° of that total. While walking on difficult terrain the head tended to maintain the change in pitch, while the eyes adjusted to retain exploratory capacity (‘t Hart and Einhäuser, 2012). Other activities that have shown notable dissociation between eye and head movement typically involve systematic and familiar tasks where gaze jumps from one point of action to another; examples include meal prep (Land, 2004) and car driving, where head angle is highly correlated with car angle, while eye-in-head rotation is uncorrelated (Land and Tatler, 2001).

Other dissociations between eye and head movements have been reported in the literature. During conversations, eye movements are minimally correlated to head movement (Collins and Barnes, 1999; Chen et al., 2012; Vrzakova et al., 2016; Grange et al., 2018; Hadley et al., 2019). Gaze behavior also plays a role in balance through the Vestibular-Ocular Reflex. The Vestibular-Ocular Reflex is the activation of eye muscles to maintain their orientation in response to head movements and tilts. To accomplish this, the eyes keep their visual orientation by moving in an equal and opposite direction to the movement of the head, with the aid of the vestibular system (Glover, 2004). As an example, during a conversation in which one person disagrees with another, that person may shake their head. To maintain eye contact while shaking, the eyes move in opposite directions to the head. Head tilts with loss of visual information have been shown to lead to increased balance difficulty (Paloski et al., 2006; Morimoto et al., 2011). In this situation, balance difficulty is observed when people tilt their heads slightly with a 30° tilt on the roll axis (Paloski et al., 2006) and can be improved when the eyes are focused on a single position (Morimoto et al., 2011).

## Hearing device intervention

### Potential areas for hearing aid intervention in communication

Modern hearing aids are equipped with directional microphones to achieve spatial gain reduction, the process which exclusively attenuates spatial locations away from targets, effectively increasing signal-to-noise ratios (SNRs) for spatially separated targets (Wouters et al., 1999; Ricketts, 2000; Gnewikow et al., 2009; Picou and Ricketts, 2017). In low-reverberation environments this strategy works well for a talker directly in front of the listener. In acoustically complex environments with multiple sound streams from different directions, the benefit of the directional microphone (relative to omnidirectional; Abrams and Kihm, 2015) requires the listener to point their head toward a stationary target (Hornsby and Ricketts, 2007), within 30° maximum. Outside this range, speech intelligibility decreases (Kuk et al., 2005), leading to the expectation that listeners will dutifully orient their head to the source of interest. Listeners with directional microphones, however, have difficulty identifying the new location of interest for large angles, take a longer amount of time to turn the head to the target, and make more complex “searching” movements (Brimijoin et al., 2014).

In general, the strongest directional effect in modern hearing aids can be found directly in front of the listener due to more aggressive microphone strategies at the front. However, studies have shown that the angle of greatest “head-turn advantage” for most people is 30° (Grange et al., 2018), with the implication that rather than pointing the head directly at a target sound source, one should turn 30° from the location of the source for optimal listening. This observation is likely due to the introduction of interaural differences that would otherwise be minimized when directing the head toward the sound source (Perrott et al., 1987). One might expect hearing aids that account for human head shifts to this angle may have improved audibility relative to other hearing aids, and indeed, greater amount of head turn advantage has been found for monaural vs. binaural hearing aids; however, there is evidence that directional microphones alone provide more of a benefit to hearing than such head-turn advantages (Ricketts, 2000). Whereas there are still avenues to improve signal processing of the device and target localization accuracy via acoustic analyses, hearing aids may benefit immensely from knowing how the listener is moving their head and interacting with the environment.

### Potential hearing-related interventions for balance

Understanding how the head is involved in stabilization and balance is important for anticipating and monitoring falls. Many individuals with hearing-loss over the age of 65 years are also at risk of falls resulting in injury (Lin et al., 2011; Lin and Ferrucci, 2012). Thus, it is worth considering how hearing devices may support these individuals beyond amplification.

The evidence for improved standing balance with the use of prosthetic hearing devices is somewhat mixed (for review, see Borsetto et al., 2021). It has been argued that hearing aids improve perception of the environment, reintroduce lost cues, and therefore improve balance and reduce fall risk (Agmon et al., 2017; Campos et al., 2018). Rumalla et al. (2015) asked a cohort of older participants (65+ years) with hearing aid experience to stand on a foam pad with feet together or on firm ground in tandem Romberg stance (heel-to-toe), in the presence of white noise, and measured the time duration before a controlled fall was observed (30 s ceiling). During the condition when hearing aids were worn, participants maintained standing balance significantly longer in both stance conditions than in the unaided condition. In a similar experiment, Vitkovic et al. (2016) showed a significant interaction between conditions of aided (on, off) and the presence of sound (on, off) but not a main effect of aided itself. Other studies showing a benefit of hearing aids on balance and fall reduction include those by Negahban et al. (2017) and Mahmoudi et al. (2019). On the other side of the argument, Kowalewski et al. (2018), while measuring the number of steps needed to regain balance after foot perturbation, showed hearing loss to be a significant factor, but the deficit was not restored with the use of hearing aids. And finally, data from a large-scale study that tested the effect of hearing aid use on gait speed and the Short Physical Performance Battery did not show an effect of hearing aid use in individuals with moderate hearing loss (*N* = 829) and those with greater hearing loss (*N* = 453; Chen et al., 2015).

One study with cochlear implant users showed a benefit on postural stability when devices were turn on compared to off (Buchman et al., 2004), but the evidence for cochlear implant use improving gait measures is mixed. One small pilot study in cochlear implant users (*N* = 3), showed that participants with bilateral caloric areflexia (severe vestibular impairment), had a modified gait cycle including increased stride time and decreased stride length when implant users had their devices turned on or were listening to music through the device, compared to the devices turned off (Hallemans et al., 2017). Conversely, Weaver et al. (2017) compared a number of gait cycle related parameters for hearing aid (*N* = 13) and cochlear implant users (*N* = 12), with their devices turned on or turned off, and failed to find significant differences between the two conditions. The authors of that study did however note that a subset of individuals exhibited marked improvement in balance metrics with devices on. These studies, and in the context of multiple studies that have shown improved standing balance in the presence of auditory cues, support the hypothesis that some individuals more than others use auditory cues for spatial awareness which can contribute to improved balance, and individuals with that listening profile are most likely to see a benefit with the use of hearing devices.

To further understand the utility of those perceptual abilities, studies of balance and hearing aid use is an area where an individualized approach might factor into future clinical decisions. That is, if aids are known to influence balance, either negatively or positively for an individual, knowing the specific factors that contribute to this outcome could be taken into consideration before fitting aids to certain at-risk populations. Finally, whereas hearing aids may provide more natural awareness of the environment and possibly mitigate fall risk, they may also play a critical role in alerting others of falls or warn the user of imminent falls. Current devices have the capability to monitor movements such as step counts, falls, or sudden impacts with the use of inertial sensors (Fabry and Bhowmik, 2021; Rahme et al., 2021). To further maximize the utility of these sensors and the information they provide, it is essential to better understand the predictive power of the behavioral patterns that head-body interactions represent.

### Final considerations for future hearing aid designs

The above sections have outlined the fundamental kinematics of head movement, relationship to between head movement and active listening and spatial awareness, communication, head-body interactions, and reviewed movement differences for individuals with hearing-loss. As continued investigation builds the knowledge base connecting head movement patterns to optimized hearing strategies, the translation to listening devices will grow. Devices, specifically hearing aids and cochlear implants, situated with bilateral point sources co-located with the ears, are well positioned to provide the necessary biometric feedback. It is realistic to speculate that future devices will have the capacity to register head movement relative to instrument-identified target location, to provide a running computation of degree and direction of alignment or misalignment with target sources that can quickly adapt to the listeners environment. This process will require dynamic signal processing by the device in response to changes in head position, target location, and importantly, the ability to accurately target primary acoustic features. Such devices will also need to operate with a fundamental knowledge of conversational dynamics to determine if body or acoustic changes warrant change to target focus. From a macro-perspective, advances in this direction will require a shift in perspective from current validation measures that focus almost exclusively on tests of speech reception to more varied measures that investigate perception of spatial awareness.

## Author contributions

All authors listed have made a substantial, direct, and intellectual contribution to the work and approved it for publication.

## Funding

This work was partially supported by Sonova Corporation in Stäfa Switzerland, University of South Florida School of Aging Studies, and Auditory and Speech Sciences Laboratory. The funder was not involved in the study design, collection, analysis, interpretation of data, the writing of this article or the decision to submit it for publication.

## Conflict of interest

The authors declare that the research was conducted in the absence of any commercial or financial relationships that could be construed as a potential conflict of interest.

## Publisher’s note

All claims expressed in this article are solely those of the authors and do not necessarily represent those of their affiliated organizations, or those of the publisher, the editors and the reviewers. Any product that may be evaluated in this article, or claim that may be made by its manufacturer, is not guaranteed or endorsed by the publisher.

## APPENDIX

Further reading

## References

[ref1] Abrams H. B. Kihm J. (2015). An introduction to MarkeTrak IX: a new baseline for the hearing aid market. Hear. Rev. 22:16.

[ref2] Agmon M. Lavie L. Doumas M. (2017). The association between hearing loss, postural control, and mobility in older adults: a systematic review. J. Am. Acad. Audiol. 28, 575–588. doi: 10.3766/jaaa.16044 28590900

[ref3] Ahrens A. Lund K. D. Marschall M. Dau T. (2019). Sound source localization with varying amount of visual information in virtual reality. PLoS One 14:e0214603. doi: 10.1371/journal.pone.0214603 30925174 PMC6440636

[ref4] Altorfer A. Jossen S. Würmle O. Käsermann M. L. Foppa K. Zimmermann H. (2000). Measurement and meaning of head movements in everyday face-to-face communicative interaction. Behav. Res. Methods Instrum. Comput. J. Psychon. Soc. Inc 32, 17–32. doi: 10.3758/bf03200785 10758661

[ref5] Anderson P. W. Zahorik P. (2014). Auditory/visual distance estimation: accuracy and variability. Front. Psychol. 5:1097. doi: 10.3389/fpsyg.2014.01097 25339924 PMC4188027

[ref6] Andonova E. Taylor H. A. (2012). Nodding in dis/agreement: a tale of two cultures. Cogn. Process. 13, S79–S82. doi: 10.1007/s10339-012-0472-x 22915258

[ref7] Anton K. Ernst A. Basta D. (2021). A static sound source can improve postural stability during walking. J. Vestib. Res. Equilib. Orientat. 31, 143–149. doi: 10.3233/VES-200015 33492257

[ref8] Archer-Boyd A. W. Holman J. A. Brimijoin W. O. (2018). The minimum monitoring signal-to-noise ratio for off-axis signals and its implications for directional hearing aids. Hear. Res. 357, 64–72. doi: 10.1016/j.heares.2017.11.011 29223929 PMC5759949

[ref9] Archer-Boyd A. W. Whitmer W. M. Brimijoin W. O. Soraghan J. J. (2015). Biomimetic direction of arrival estimation for resolving front-back confusions in hearing aids. J. Acoust. Soc. Am. 137:EL360-366. doi: 10.1121/1.4918297 25994734 PMC4556066

[ref10] Assländer L. Peterka R. J. (2014). Sensory reweighting dynamics in human postural control. J. Neurophysiol. 111, 1852–1864. doi: 10.1152/jn.00669.2013 24501263 PMC4044370

[ref11] Baird J. L. Van Emmerik R. E. A. (2009). Young and older adults use different strategies to perform a standing turning task. Clin. Biomech. 24, 826–832. doi: 10.1016/j.clinbiomech.2009.08.006 19766364

[ref12] Bernieri F. J. (1988). Coordinated movement and rapport in teacher-student interactions. J. Nonverbal Behav. 12, 120–138. doi: 10.1007/BF00986930

[ref13] Blauert J. (2001). Spatial hearing: the psychophysics of human sound localization. Cambridge, Massachusetts: MIT Press.

[ref14] Bogduk N. Mercer S. (2000). Biomechanics of the cervical spine. I: Normal kinematics. Clin. Biomech. (Bristol, Avon) 15, 633–648. doi: 10.1016/s0268-0033(00)00034-6 10946096

[ref15] Boker S. M. Cohn J. F. Theobald B.-J. Matthews I. Brick T. R. Spies J. R. (2009). Effects of damping head movement and facial expression in dyadic conversation using real-time facial expression tracking and synthesized avatars. Philos. Trans. R. Soc. Lond. B Biol. Sci. 364, 3485–3495. doi: 10.1098/rstb.2009.0152 19884143 PMC2781890

[ref16] Borsetto D. Corazzi V. Franchella S. Bianchini C. Pelucchi S. Obholzer R.. (2021). The influence of hearing aids on balance control: a systematic review. Audiol. Neurootol. 26, 209–217. doi: 10.1159/000511135 33316800

[ref17] Bousmalis K. Mehu M. Pantic M. (2013). Towards the automatic detection of spontaneous agreement and disagreement based on nonverbal behaviour: a survey of related cues, databases, and tools. Image Vis. Comput. 31, 203–221. doi: 10.1016/j.imavis.2012.07.003

[ref18] Brimijoin W. O. McShefferty D. Akeroyd M. A. (2010). Auditory and visual orienting responses in listeners with and without hearing-impairment. J. Acoust. Soc. Am. 127, 3678–3688. doi: 10.1121/1.3409488 20550266 PMC4338612

[ref19] Brimijoin W. O. McShefferty D. Akeroyd M. A. (2012). Undirected head movements of listeners with asymmetrical hearing impairment during a speech-in-noise task. Hear. Res. 283, 162–168. doi: 10.1016/j.heares.2011.10.009 22079774 PMC3315013

[ref20] Brimijoin W. O. Whitmer W. M. McShefferty D. Akeroyd M. A. (2014). The effect of hearing aid microphone mode on performance in an auditory orienting task. Ear Hear. 35, e204–e212. doi: 10.1097/AUD.0000000000000053 25148290 PMC4232295

[ref21] Brodie M. A. Lovell N. H. Canning C. G. Menz H. B. Delbaere K. Redmond S. J.. (2014). Gait as a biomarker? Accelerometers reveal that reduced movement quality while walking is associated with Parkinson’s disease, ageing and fall risk. Annu. Int. Conf. IEEE Eng. Med. Biol. Soc. 2014, 5968–5971. doi: 10.1109/EMBC.2014.6944988 25571356

[ref22] Bronkhorst A. W. Houtgast T. (1999). Auditory distance perception in rooms. Nature 397, 517–520. doi: 10.1038/17374 10028966

[ref23] Brown A. D. Beemer B. T. Greene N. T. Argo T. Meegan G. D. Tollin D. J. (2015a). Effects of active and passive hearing protection devices on sound source localization, speech recognition, and tone detection. PLoS One 10:e0136568. doi: 10.1371/journal.pone.0136568 26313145 PMC4551850

[ref24] Brown A. D. Stecker G. C. Tollin D. J. (2015b). The precedence effect in sound localization. J. Assoc. Res. Otolaryngol. 16, 1–28. doi: 10.1007/s10162-014-0496-2 25479823 PMC4310855

[ref25] Bruce H. Aponte D. St-Onge N. Phillips N. Gagné J.-P. Li K. Z. H. (2019). The effects of age and hearing loss on dual-task balance and listening. J. Gerontol. B Psychol. Sci. Soc. Sci. 74, 275–283. doi: 10.1093/geronb/gbx047 28486677 PMC6327658

[ref26] Brungart D. S. Cohen J. I. Zion D. Romigh G. (2017). The localization of non-individualized virtual sounds by hearing impaired listeners. J. Acoust. Soc. Am. 141:2870. doi: 10.1121/1.4979462 28464685

[ref27] Buchman C. A. Joy J. Hodges A. Telischi F. F. Balkany T. J. (2004). Vestibular effects of cochlear implantation. Laryngoscope 114, 1–22. doi: 10.1097/00005537-200410001-00001 15454752

[ref28] Cai T. Rakerd B. Hartmann W. M. (2015). Computing interaural differences through finite element modeling of idealized human heads. J. Acoust. Soc. Am. 138, 1549–1560. doi: 10.1121/1.4927491 26428792 PMC4575315

[ref29] Campos J. Ramkhalawansingh R. Pichora-Fuller M. K. (2018). Hearing, self-motion perception, mobility, and aging. Hear. Res. 369, 42–55. doi: 10.1016/j.heares.2018.03.025 29661612

[ref30] Carlile S. Leung J. (2016). The perception of auditory motion. Trends Hear. 20:2331216516644254. doi: 10.1177/2331216516644254 27094029 PMC4871213

[ref31] Chen D. S. Betz J. Yaffe K. Ayonayon H. N. Kritchevsky S. Martin K. R.. (2015). Association of hearing impairment with declines in physical functioning and the risk of disability in older adults. J. Gerontol. A Biol. Sci. Med. Sci. 70, 654–661. doi: 10.1093/gerona/glu207 25477427 PMC4400396

[ref32] Chen L. L. Lee D. Fukushima K. Fukushima J. (2012). Submovement composition of head movement. PLoS One 7:e47565. doi: 10.1371/journal.pone.0047565 23139749 PMC3489904

[ref33] Cherry E. C. (1953). Some experiments on the recognition of speech, with one and with two ears. J. Acoust. Soc. Am. 25, 975–979. doi: 10.1121/1.1907229

[ref34] Ching R. (2007). Relationship between head mass and circumference in human adults. Available at: https://www.semanticscholar.org/paper/Relationship-Between-Head-Mass-and-Circumference-in-Ching/f06ec5c1eddaa7f731d1255b16ae7075b0d887cc (Accessed 17 January 2023).

[ref35] Collins C. J. Barnes G. R. (1999). Independent control of head and gaze movements during head-free pursuit in humans. J. Physiol. 515, 299–314. doi: 10.1111/j.1469-7793.1999.299ad.x 9925900 PMC2269145

[ref36] Cornwell T. Woodward J. Wu M. Jackson B. Souza P. Siegel J.. (2020). Walking with ears: altered auditory feedback impacts gait step length in older adults. Front. Sports Act. Living 2:38. doi: 10.3389/fspor.2020.00038 33345030 PMC7739652

[ref37] Cotzin M. Dallenbach K. M. (1950). “Facial vision:” the role of pitch and loudness in the perception of obstacles by the blind. Am. J. Psychol. 63, 485–515. 14790019

[ref38] Cromwell R. L. (2003). Movement strategies for head stabilization during incline walking. Gait Posture 17, 246–253. doi: 10.1016/s0966-6362(02)00094-2 12770638

[ref39] Cromwell R. Schurter J. Shelton S. Vora S. (2004). Head stabilization strategies in the sagittal plane during locomotor tasks. Physiother. Res. Int. J. Res. Clin. Phys. Ther. 9, 33–42. doi: 10.1002/pri.298 15132026

[ref40] David M. Lavandier M. Grimault N. Oxenham A. J. (2017). Discrimination and streaming of speech sounds based on differences in interaural and spectral cues. J. Acoust. Soc. Am. 142:1674. doi: 10.1121/1.5003809 28964066 PMC5617732

[ref41] de Souza Melo R. (2017). Ampleness of head movements of children and adolescents with sensorineural hearing loss. Int. J. Pediatr. Otorhinolaryngol. 93, 133–140. doi: 10.1016/j.ijporl.2016.12.015 28109485

[ref42] Deviterne D. Gauchard G. C. Jamet M. Vançon G. Perrin P. P. (2005). Added cognitive load through rotary auditory stimulation can improve the quality of postural control in the elderly. Brain Res. Bull. 64, 487–492. doi: 10.1016/j.brainresbull.2004.10.007 15639544

[ref43] Diehl M. D. Pidcoe P. E. (2011). The role of head-in-space stability on stepping reactions in young and elderly adults. Physiother. Theory Pract. 27, 337–344. doi: 10.3109/09593985.2010.512357 20812855

[ref44] Dozza M. Horak F. B. Chiari L. (2007). Auditory biofeedback substitutes for loss of sensory information in maintaining stance. Exp. Brain Res. 178, 37–48. doi: 10.1007/s00221-006-0709-y 17021893

[ref45] Drennan W. R. Gatehouse S. Howell P. Van Tasell D. Lund S. (2005). Localization and speech-identification ability of hearing-impaired listeners using phase-preserving amplification. Ear Hear. 26, 461–472. doi: 10.1097/01.aud.0000179690.30137.21 16230896

[ref46] Duran N. D. Fusaroli R. (2017). Conversing with a devil’s advocate: interpersonal coordination in deception and disagreement. PLoS One 12:e0178140. doi: 10.1371/journal.pone.0178140 28574996 PMC5456047

[ref47] Dvorak J. Antinnes J. A. Panjabi M. Loustalot D. Bonomo M. (1992). Age and gender related normal motion of the cervical spine. Spine 17, S393–S398. doi: 10.1097/00007632-199210001-00009 1440033

[ref48] Easton R. D. Greene A. J. DiZio P. Lackner J. R. (1998). Auditory cues for orientation and postural control in sighted and congenitally blind people. Exp. Brain Res. 118, 541–550. doi: 10.1007/s002210050310 9504849

[ref49] Fabry D. A. Bhowmik A. K. (2021). Improving speech understanding and monitoring health with hearing aids using artificial intelligence and embedded sensors. Semin. Hear. 42, 295–308. doi: 10.1055/s-0041-1735136 34594091 PMC8463124

[ref50] Ferrario V. F. Sforza C. Serrao G. Grassi G. Mossi E. (2002). Active range of motion of the head and cervical spine: a three-dimensional investigation in healthy young adults. J. Orthop. Res. Off. Publ. Orthop. Res. Soc. 20, 122–129. doi: 10.1016/S0736-0266(01)00079-1 11853078

[ref51] Fitts P. M. (1954). The information capacity of the human motor system in controlling the amplitude of movement. J. Exp. Psychol. 47, 381–391. 13174710

[ref52] Franchak J. M. McGee B. Blanch G. (2021). Adapting the coordination of eyes and head to differences in task and environment during fully-mobile visual exploration. PLoS One 16:e0256463. doi: 10.1371/journal.pone.0256463 34415981 PMC8378697

[ref53] Franssen N. V. (1960). Some considerations on the mechanism of directional hearing. The Netherlands: Doctoral Dissertation. Technische Hogeschool, Delft.

[ref54] Gandemer L. Parseihian G. Kronland-Martinet R. Bourdin C. (2014). The influence of horizontally rotating sound on standing balance. Exp. Brain Res. 232, 3813–3820. doi: 10.1007/s00221-014-4066-y 25146572

[ref55] Gandemer L. Parseihian G. Kronland-Martinet R. Bourdin C. (2017). Spatial cues provided by sound improve postural stabilization: evidence of a spatial auditory map? Front. Neurosci. 11:357. doi: 10.3389/fnins.2017.00357 28694770 PMC5483472

[ref56] Gessa E. Giovanelli E. Spinella D. Verdelet G. Farnè A. Frau G. N.. (2022). Spontaneous head-movements improve sound localization in aging adults with hearing loss. Front. Hum. Neurosci. 16:1026056. doi: 10.3389/fnhum.2022.1026056 36310849 PMC9609159

[ref57] Gibson J. J. (1966). The senses considered as perceptual systems. Boston: Houghton Mifflin.

[ref58] Glover J. C. (2004). “Vestibular System” in Encyclopedia of neuroscience. ed. Squire L. R. (Oxford: Academic Press), 127–132.

[ref59] Gnewikow D. Ricketts T. Bratt G. W. Mutchler L. C. (2009). Real-world benefit from directional microphone hearing aids. J. Rehabil. Res. Dev. 46, 603–618. doi: 10.1682/jrrd.2007.03.0052 19882494

[ref60] Graf W. de Waele C. Vidal P. P. (1995). Functional anatomy of the head-neck movement system of quadrupedal and bipedal mammals. J. Anat. 186, 55–74. 7649818 PMC1167272

[ref61] Grange J. A. Culling J. F. (2016a). Head orientation benefit to speech intelligibility in noise for cochlear implant users and in realistic listening conditions. J. Acoust. Soc. Am. 140:4061. doi: 10.1121/1.4968515 28039996

[ref62] Grange J. A. Culling J. F. (2016b). The benefit of head orientation to speech intelligibility in noise. J. Acoust. Soc. Am. 139, 703–712. doi: 10.1121/1.4941655 26936554

[ref63] Grange J. A. Culling J. F. Bardsley B. Mackinney L. I. Hughes S. E. Backhouse S. S. (2018). Turn an ear to hear: how hearing-impaired listeners can exploit head orientation to enhance their speech intelligibility in Noisy social settings. Trends Hear. 22:2331216518802701. doi: 10.1177/2331216518802701 30334495 PMC6196611

[ref64] Hadar U. Steiner T. J. Grant E. C. Rose F. C. (1983). Kinematics of head movements accompanying speech during conversation. Hum. Mov. Sci. 2, 35–46. doi: 10.1016/0167-9457(83)90004-0

[ref65] Hadley L. V. Brimijoin W. O. Whitmer W. M. (2019). Speech, movement, and gaze behaviours during dyadic conversation in noise. Sci. Rep. 9:10451. doi: 10.1038/s41598-019-46416-0 31320658 PMC6639257

[ref66] Hale J. Ward J. A. Buccheri F. Oliver D. Hamilton A. F. (2020). Are you on my wavelength? Interpersonal coordination in dyadic conversations. J. Nonverbal Behav. 44, 63–83. doi: 10.1007/s10919-019-00320-3 32189820 PMC7054373

[ref67] Hall J. A. Horgan T. G. Murphy N. A. (2019). Nonverbal communication. Annu. Rev. Psychol. 70, 271–294. doi: 10.1146/annurev-psych-010418-103145 30256720

[ref68] Hallemans A. Mertens G. Van de Heyning P. Van Rompaey V. (2017). Playing music may improve the gait pattern in patients with bilateral caloric Areflexia wearing a Cochlear implant: results from a pilot study. Front. Neurol. 8:404. doi: 10.3389/fneur.2017.00404 28861034 PMC5562687

[ref69] Heffner R. S. (1997). Comparative study of sound localization and its anatomical correlates in mammals. Acta Otolaryngol. Suppl. 532, 46–53. doi: 10.3109/00016489709126144 9442844

[ref70] Hendrikse M. M. E. Llorach G. Grimm G. Hohmann V. (2018). Influence of visual cues on head and eye movements during listening tasks in multi-talker audiovisual environments with animated characters. Speech Commun. 101, 70–84. doi: 10.1016/j.specom.2018.05.008

[ref71] Hendrikse M. M. E. Llorach G. Hohmann V. Grimm G. (2019). Movement and gaze behavior in virtual audiovisual listening environments resembling everyday life. Trends Hear. 23:2331216519872362. doi: 10.1177/2331216519872362 32516060 PMC6732870

[ref72] Heylen D. (2008). “Listening Heads” in Modeling communication with robots and virtual humans. eds. Wachsmuth I. Knoblich G. (Berlin, Heidelberg: Springer Berlin Heidelberg), 241–259.

[ref73] Hirahara T. Sagara H. Toshima I. Otani M. (2010). Head movement during head-related transfer function measurements. Acoust. Sci. Technol. 31, 165–171. doi: 10.1250/ast.31.165

[ref74] Hirasaki E. Kubo T. Nozawa S. Matano S. Matsunaga T. (1993). Analysis of head and body movements of elderly people during locomotion. Acta Otolaryngol. Suppl. 501, 25–30. doi: 10.3109/00016489309126208 8447221

[ref75] Hoffmann E. R. Chan A. H. S. Heung P. T. (2017). Head rotation movement times. Hum. Factors 59, 986–994. doi: 10.1177/0018720817701000 28796975

[ref76] Hornsby B. W. Y. Ricketts T. A. (2007). Effects of noise source configuration on directional benefit using symmetric and asymmetric directional hearing aid fittings. Ear Hear. 28, 177–186. doi: 10.1097/AUD.0b013e3180312639 17496669

[ref77] Hüg M. X. Bermejo F. Tommasini F. C. Di Paolo E. A. (2022). Effects of guided exploration on reaching measures of auditory peripersonal space. Front. Psychol. 13:983189. doi: 10.3389/fpsyg.2022.983189 36337523 PMC9632294

[ref78] Humes L. E. Dubno J. R. (2010). “Factors affecting speech understanding in older adults” in The aging auditory system. eds. Gordon-Salant S. Frisina R. D. Popper A. N. Fay R. R. (New York, NY: Springer New York), 211–257.

[ref79] Inokuchi H. Tojima M. Mano H. Ishikawa Y. Ogata N. Haga N. (2015). Neck range of motion measurements using a new three-dimensional motion analysis system: validity and repeatability. Eur. Spine J. off. Publ. Eur. Spine Soc. Eur. Spinal deform. Soc. Eur. Sect. Cerv. Spine Res. Soc. 24, 2807–2815. doi: 10.1007/s00586-015-3913-2 25847728

[ref80] Jelfs S. Culling J. F. Lavandier M. (2011). Revision and validation of a binaural model for speech intelligibility in noise. Hear. Res. 275, 96–104. doi: 10.1016/j.heares.2010.12.005 21156201

[ref81] Kanegaonkar R. G. Amin K. Clarke M. (2012). The contribution of hearing to normal balance. J. Laryngol. Otol. 126, 984–988. doi: 10.1017/S002221511200179X 22906584

[ref82] Katz J. Burkard R. F. Medwetsky L. (2002). Handbook of clinical audiology. Lippincott Williams & Wilkins Available at: https://books.google.com/books?id=Aj6nVIegE6AC.

[ref83] Kavanagh J. Barrett R. Morrison S. (2006). The role of the neck and trunk in facilitating head stability during walking. Exp. Brain Res. 172, 454–463. doi: 10.1007/s00221-006-0353-6 16489437

[ref84] Keidser G. O’Brien A. Hain J.-U. McLelland M. Yeend I. (2009). The effect of frequency-dependent microphone directionality on horizontal localization performance in hearing-aid users. Int. J. Audiol. 48, 789–803. doi: 10.3109/14992020903036357 19951147

[ref85] Keidser G. Rohrseitz K. Dillon H. Hamacher V. Carter L. Rass U.. (2006). The effect of multi-channel wide dynamic range compression, noise reduction, and the directional microphone on horizontal localization performance in hearing aid wearers. Int. J. Audiol. 45, 563–579. doi: 10.1080/14992020600920804 17062498

[ref86] Kendon A. (1970). Movement coordination in social interaction: some examples described. Acta Psychol. (Amst) 32, 101–125. doi: 10.1016/0001-6918(70)90094-6 5444439

[ref87] Kendon A. (2002). Some uses of the head shake. Gesture 2, 147–182. doi: 10.1075/gest.2.2.03ken

[ref88] Keshner E. A. (2004). Head-trunk coordination in elderly subjects during linear anterior-posterior translations. Exp. Brain Res. 158, 213–222. doi: 10.1007/s00221-004-1893-2 15071712

[ref89] Kock W. E. (1950). Binaural localization and masking. J. Acoust. Soc. Am. 22, 801–804. doi: 10.1121/1.1906692

[ref90] Kolarik A. J. Cirstea S. Pardhan S. Moore B. C. J. (2014). A summary of research investigating echolocation abilities of blind and sighted humans. Hear. Res. 310, 60–68. doi: 10.1016/j.heares.2014.01.010 24524865

[ref91] Kolarik A. J. Moore B. C. J. Zahorik P. Cirstea S. Pardhan S. (2016). Auditory distance perception in humans: a review of cues, development, neuronal bases, and effects of sensory loss. Atten. Percept. Psychophys. 78, 373–395. doi: 10.3758/s13414-015-1015-1 26590050 PMC4744263

[ref92] Kondo H. M. Pressnitzer D. Toshima I. Kashino M. (2012). Effects of self-motion on auditory scene analysis. Proc. Natl. Acad. Sci. U. S. A. 109, 6775–6780. doi: 10.1073/pnas.1112852109 22493250 PMC3340062

[ref93] Kopčo N. Shinn-Cunningham B. G. (2011). Effect of stimulus spectrum on distance perception for nearby sources. J. Acoust. Soc. Am. 130, 1530–1541. doi: 10.1121/1.3613705 21895092 PMC3188969

[ref94] Kousidis S. Malisz Z. Wagner P. Schlangen D. (2013). Exploring annotation of head gesture forms in spontaneous human interaction. Available at: https://www.semanticscholar.org/paper/Exploring-annotation-of-head-gesture-forms-in-human-Kousidis-Malisz/48945438f28db9bdf2d75cb9878058d86b6d4acd (Accessed 17 January 2023).

[ref95] Kowalewski V. Patterson R. Hartos J. Bugnariu N. (2018). Hearing loss contributes to balance difficulties in both younger and older adults. J. Prev. Med. 3:12. doi: 10.21767/2572-5483.100033 PMC6017998 29951645

[ref96] Kuk F. Keenan D. Lau C.-C. Ludvigsen C. (2005). Performance of a fully adaptive directional microphone to signals presented from various azimuths. J. Am. Acad. Audiol. 16, 333–347. doi: 10.3766/jaaa.16.6.2 16178405

[ref97] Kunin M. Osaki Y. Cohen B. Raphan T. (2007). Rotation axes of the head during positioning, head shaking, and locomotion. J. Neurophysiol. 98, 3095–3108. doi: 10.1152/jn.00764.2007 17898142

[ref98] Kuo F.-C. Hong C.-Z. Liau B.-Y. (2014). Kinematics and muscle activity of the head, lumbar and knee joints during 180° turning and sitting down task in older adults. Clin. Biomech. (Bristol, Avon) 29, 14–20. doi: 10.1016/j.clinbiomech.2013.10.020 24239023

[ref99] Lakin J. L. Jefferis V. E. Cheng C. M. Chartrand T. L. (2003). The chameleon effect as social glue: evidence for the evolutionary significance of nonconscious mimicry. J. Nonverbal Behav. 27, 145–162. doi: 10.1023/A:1025389814290

[ref100] Land M. F. (2004). The coordination of rotations of the eyes, head and trunk in saccadic turns produced in natural situations. Exp. Brain Res. 159, 151–160. doi: 10.1007/s00221-004-1951-9 15221164

[ref101] Land M. F. Tatler B. W. (2001). Steering with the head. The visual strategy of a racing driver. Curr. Biol. 11, 1215–1220. doi: 10.1016/s0960-9822(01)00351-7 11516955

[ref102] Latif N. Barbosa A. V. Vatikiotis-Bateson E. Castelhano M. S. Munhall K. G. (2014). Movement coordination during conversation. PLoS One 9:e105036. doi: 10.1371/journal.pone.0105036 25119189 PMC4132081

[ref103] Letz R. Gerr F. (1995). Standing steadiness measurements: empirical selection of testing protocol and outcome measures. Neurotoxicol. Teratol. 17, 611–616. doi: 10.1016/0892-0362(95)00023-2 8747742

[ref104] Li L. Simonsick E. M. Ferrucci L. Lin F. R. (2013). Hearing loss and gait speed among older adults in the United States. Gait Posture 38, 25–29. doi: 10.1016/j.gaitpost.2012.10.006 23177614 PMC3845825

[ref105] Lin F. R. Ferrucci L. (2012). Hearing loss and falls among older adults in the United States. Arch. Intern. Med. 172, 369–371. doi: 10.1001/archinternmed.2011.728 22371929 PMC3518403

[ref106] Lin F. R. Niparko J. K. Ferrucci L. (2011). Hearing loss prevalence in the United States. Arch. Intern. Med. 171, 1851–1853. doi: 10.1001/archinternmed.2011.506 22083573 PMC3564588

[ref107] Livingstone S. R. Palmer C. (2016). Head movements encode emotions during speech and song. Emotion 16, 365–380. doi: 10.1037/emo0000106 26501928

[ref108] Lorenzi C. Gatehouse S. Lever C. (1999). Sound localization in noise in hearing-impaired listeners. J. Acoust. Soc. Am. 105, 3454–3463. doi: 10.1121/1.424672 10380669

[ref109] Lu H. Brimijoin W. O. (2022). Sound source selection based on head movements in natural group conversation. Trends Hear. 26:23312165221097788. doi: 10.1177/23312165221097789 PMC9058564 35477340

[ref110] Lu H. McKinney M. F. Zhang T. Oxenham A. J. (2021). Investigating age, hearing loss, and background noise effects on speaker-targeted head and eye movements in three-way conversations. J. Acoust. Soc. Am. 149:1889. doi: 10.1121/10.0003707 33765809

[ref111] Mahmoudi E. Basu T. Langa K. McKee M. M. Zazove P. Alexander N.. (2019). Can hearing aids delay time to diagnosis of dementia, depression, or falls in older adults? J. Am. Geriatr. Soc. 67, 2362–2369. doi: 10.1111/jgs.16109 31486068

[ref112] Maurer C. Mergner T. Bolha B. Hlavacka F. (2000). Vestibular, visual, and somatosensory contributions to human control of upright stance. Neurosci. Lett. 281, 99–102. doi: 10.1016/s0304-3940(00)00814-4 10704752

[ref113] McLachlan G. Majdak P. Reijniers J. Mihocic M. Peremans H. (2023). Dynamic spectral cues do not affect human sound localization during small head movements. Front. Neurosci. 17:1027827. doi: 10.3389/fnins.2023.1027827 36816108 PMC9936143

[ref114] Mershon D. H. King L. E. (1975). Intensity and reverberation as factors in the auditory perception of egocentric distance. Percept. Psychophys. 18, 409–415. doi: 10.3758/BF03204113

[ref115] Milne J. L. Goodale M. A. Thaler L. (2014). The role of head movements in the discrimination of 2-D shape by blind echolocation experts. Atten. Percept. Psychophys. 76, 1828–1837. doi: 10.3758/s13414-014-0695-2 24874262

[ref116] Moore S. T. Hirasaki E. Raphan T. Cohen B. (2005). Instantaneous rotation axes during active head movements. J. Vestib. Res. Equilib. Orientat. 15, 73–80. doi: 10.3233/VES-2005-15203 15951621

[ref117] Morimoto H. Asai Y. Johnson E. G. Lohman E. B. Khoo K. Mizutani Y.. (2011). Effect of oculo-motor and gaze stability exercises on postural stability and dynamic visual acuity in healthy young adults. Gait Posture 33, 600–603. doi: 10.1016/j.gaitpost.2011.01.016 21334899

[ref118] Munhall K. G. Jones J. A. Callan D. E. Kuratate T. Vatikiotis-Bateson E. (2004). Visual prosody and speech intelligibility: head movement improves auditory speech perception. Psychol. Sci. 15, 133–137. doi: 10.1111/j.0963-7214.2004.01502010.x 14738521

[ref119] Negahban H. Ali B. C. Nassadj G. (2017). Effect of hearing aids on static balance function in elderly with hearing loss. Gait Posture 58, 126–129. doi: 10.1016/j.gaitpost.2017.07.112 28772132

[ref120] Nguyen A. K. D. Simard-Meilleur A. A. Berthiaume C. Godbout R. Mottron L. (2012). Head circumference in Canadian male adults: development of a normalized chart. Int. J. Morphol. 30, 1474–1480. doi: 10.4067/S0717-95022012000400033

[ref121] Novelia A. (2015). On the configuration manifold of a rigid body subject to Listing’s constraint. University of California, Berkeley:Master’s Thesis.

[ref122] Otte R. J. Agterberg M. J. H. Van Wanrooij M. M. Snik A. F. M. Van Opstal A. J. (2013). Age-related hearing loss and ear morphology affect vertical but not horizontal sound-localization performance. J. Assoc. Res. Otolaryngol. 14, 261–273. doi: 10.1007/s10162-012-0367-7 23319012 PMC3660912

[ref123] Paloski W. H. Wood S. J. Feiveson A. H. Black F. O. Hwang E. Y. Reschke M. F. (2006). Destabilization of human balance control by static and dynamic head tilts. Gait Posture 23, 315–323. doi: 10.1016/j.gaitpost.2005.04.009 15961313

[ref124] Papadopoulos T. Edwards D. S. Rowan D. Allen R. (2011). Identification of auditory cues utilized in human echolocation—objective measurement results. Biomed. Signal Process. Control 6, 280–290. doi: 10.1016/j.bspc.2011.03.005

[ref125] Paquette C. Paquet N. Fung J. (2006). Aging affects coordination of rapid head motions with trunk and pelvis movements during standing and walking. Gait Posture 24, 62–69. doi: 10.1016/j.gaitpost.2005.07.001 16098745

[ref126] Park S. H. Lee K. Lockhart T. Kim S. (2011). Effects of sound on postural stability during quiet standing. J. Neuroeng. Rehabil. 8:67. doi: 10.1186/1743-0003-8-67 22168248 PMC3275494

[ref127] Park M. S. Moon S.-H. Lee H.-M. Kim T.-H. Oh J. K. Nam J. H.. (2014). Age-related changes in cervical sagittal range of motion and alignment. Glob. Spine J. 4, 151–156. doi: 10.1055/s-0034-1378140 PMC4111948 25083355

[ref128] Parseihian G. Jouffrais C. Katz B. F. G. (2014). Reaching nearby sources: comparison between real and virtual sound and visual targets. Front. Neurosci. 8:269. doi: 10.3389/fnins.2014.00269 25228855 PMC4151089

[ref129] Perrott D. R. Ambarsoom H. Tucker J. (1987). Changes in head position as a measure of auditory localization performance: auditory psychomotor coordination under monaural and binaural listening conditions. J. Acoust. Soc. Am. 82, 1637–1645. doi: 10.1121/1.395155 3693705

[ref130] Peterka R. J. (2018). Sensory integration for human balance control. Handb. Clin. Neurol. 159, 27–42. doi: 10.1016/B978-0-444-63916-5.00002-1 30482320

[ref131] Picou E. M. Ricketts T. A. (2017). How directional microphones affect speech recognition, listening effort and localisation for listeners with moderate-to-severe hearing loss. Int. J. Audiol. 56, 909–918. doi: 10.1080/14992027.2017.1355074 28738747

[ref132] Pollack I. Rose M. (1967). Effect of head movement on the localization of sounds in the equatorial plane. Percept. Psychophys. 2, 591–596. doi: 10.3758/BF03210274

[ref133] Rahme M. Folkeard P. Scollie S. (2021). Evaluating the accuracy of step tracking and fall detection in the Starkey Livio artificial intelligence hearing aids: a pilot study. Am. J. Audiol. 30, 182–189. doi: 10.1044/2020_AJA-20-00105 33284647

[ref134] Raper S. A. Soames R. W. (1991). The influence of stationary auditory fields on postural sway behaviour in man. Eur. J. Appl. Physiol. 63, 363–367. doi: 10.1007/BF00364463 1773813

[ref135] Ricketts T. (2000). The impact of head angle on monaural and binaural performance with directional and omnidirectional hearing aids. Ear Hear. 21, 318–328. doi: 10.1097/00003446-200008000-00007 10981608

[ref136] Ricketts T. A. Bentler R. Mueller H. G. (2017). Essentials of modern hearing aids: Selection, fitting, and verification. Plural Publishing, Incorporated Available at: https://books.google.com/books?id=2ddiDwAAQBAJ.

[ref137] Rojas J. A. M. Hermosilla J. A. Montero R. S. Espí P. L. L. (2009). Physical analysis of several organic signals for human echolocation: Oral vacuum pulses. Acta Acust. United Acust. 95, 325–330. doi: 10.3813/AAA.918155

[ref138] Rönnberg J. (2003). Cognition in the hearing impaired and deaf as a bridge between signal and dialogue: a framework and a model. Int. J. Audiol. 42, S68–S76. doi: 10.3109/14992020309074626 12918612

[ref139] Rönnberg J. Signoret C. Andin J. Holmer E. (2022). The cognitive hearing science perspective on perceiving, understanding, and remembering language: the ELU model. Front. Psychol. 13:967260. doi: 10.3389/fpsyg.2022.967260 36118435 PMC9477118

[ref140] Rosenblum L. D. Gordon M. S. Jarquin L. (2000). Echolocating distance by moving and stationary listeners. Ecol. Psychol. 12, 181–206. doi: 10.1207/S15326969ECO1203_1

[ref141] Ross J. M. Balasubramaniam R. (2015). Auditory white noise reduces postural fluctuations even in the absence of vision. Exp. Brain Res. 233, 2357–2363. doi: 10.1007/s00221-015-4304-y 25953650

[ref142] Rowan D. Papadopoulos T. Edwards D. Holmes H. Hollingdale A. Evans L.. (2013). Identification of the lateral position of a virtual object based on echoes by humans. Hear. Res. 300, 56–65. doi: 10.1016/j.heares.2013.03.005 23538130

[ref143] Rumalla K. Karim A. M. Hullar T. E. (2015). The effect of hearing aids on postural stability. Laryngoscope 125, 720–723. doi: 10.1002/lary.24974 25346316

[ref144] Sanchez-Lopez R. Fereczkowski M. Neher T. Santurette S. Dau T. (2020). Robust data-driven auditory profiling towards precision audiology. Trends Hear. 24:2331216520973539. doi: 10.1177/2331216520973539 33272110 PMC7720332

[ref145] Schenkman B. N. Nilsson M. E. (2010). Human echolocation: blind and sighted persons’ ability to detect sounds recorded in the presence of a reflecting object. Perception 39, 483–501. doi: 10.1068/p6473 20514997

[ref146] Schenkman B. N. Nilsson M. E. (2011). Human echolocation: pitch versus loudness information. Perception 40, 840–852. doi: 10.1068/p6898 22128556

[ref147] Schneider B. A. Pichora-Fuller K. Daneman M. (2010). “Effects of senescent changes in audition and cognition on spoken language comprehension” in The aging auditory system. eds. Gordon-Salant S. Frisina R. D. Popper A. N. Fay R. R. (New York, NY: Springer New York), 167–210.

[ref148] Schörnich S. Wallmeier L. Gessele N. Nagy A. Schranner M. Kish D.. (2013). Psychophysics of human echolocation. Adv. Exp. Med. Biol. 787, 311–319. doi: 10.1007/978-1-4614-1590-9_35 23716237

[ref149] Seidler R. D. Bernard J. A. Burutolu T. B. Fling B. W. Gordon M. T. Gwin J. T.. (2010). Motor control and aging: links to age-related brain structural, functional, and biochemical effects. Neurosci. Biobehav. Rev. 34, 721–733. doi: 10.1016/j.neubiorev.2009.10.005 19850077 PMC2838968

[ref150] Seiwerth I. Jonen J. Rahne T. Lauenroth A. Hullar T. E. Plontke S. K.. (2020). Postural regulation and stability with acoustic input in normal-hearing subjects. HNO 68, 100–105. doi: 10.1007/s00106-020-00846-9 PMC7403163 32377779

[ref151] Sibley K. M. Beauchamp M. K. Van Ooteghem K. Straus S. E. Jaglal S. B. (2015). Using the systems framework for postural control to analyze the components of balance evaluated in standardized balance measures: a scoping review. Arch. Phys. Med. Rehabil. 96, 122–132.e29. doi: 10.1016/j.apmr.2014.06.021 25073007

[ref152] Skoglund M. A. Andersen M. Shiell M. M. Keidser G. Rank M. L. Rotger-Griful S. (2022). Comparing in-ear EOG for eye-movement estimation with eye-tracking: accuracy, calibration, and speech comprehension. Front. Neurosci. 16:873201. doi: 10.3389/fnins.2022.873201 35844213 PMC9279575

[ref153] Stecker G. C. (2018). Temporal binding of auditory spatial information across dynamic binaural events. Atten. Percept. Psychophys. 80, 14–20. doi: 10.3758/s13414-017-1436-0 29086219 PMC5735209

[ref154] Stecker G. C. Moore T. M. (2018). Reverberation enhances onset dominance in sound localization. J. Acoust. Soc. Am. 143:786. doi: 10.1121/1.5023221 29495688 PMC5805551

[ref155] Stevens M. N. Barbour D. L. Gronski M. P. Hullar T. E. (2016). Auditory contributions to maintaining balance. J. Vestib. Res. Equilib. Orientat. 26, 433–438. doi: 10.3233/VES-160599 28262648

[ref156] Stiefelhagen R. Zhu J. (2002). “CHI ‘02 extended abstracts on human factors in computing systems CHI EA ‘02” in Head orientation and gaze direction in meetings (New York, NY, USA: Association for Computing Machinery, and the Conference Chairs were Loren Terveen and Dennis Wixon), 858–859.

[ref157] Stöckel T. Jacksteit R. Behrens M. Skripitz R. Bader R. Mau-Moeller A. (2015). The mental representation of the human gait in young and older adults. Front. Psychol. 6:943. doi: 10.3389/fpsyg.2015.00943 26236249 PMC4500916

[ref158] Stoffregen T. A. Villard S. Kim C. Ito K. Bardy B. G. (2009). Coupling of head and body movement with motion of the audible environment. J. Exp. Psychol. Hum. Percept. Perform. 35, 1221–1231. doi: 10.1037/a0014251 19653760

[ref159] Stone M. Oh I. (2008). “Modeling facial expression of uncertainty in conversational animation” in Modeling communication with robots and virtual humans. eds. Wachsmuth I. Knoblich G. (Berlin, Heidelberg: Springer Berlin Heidelberg), 57–76.

[ref160] Stroffregen T. A. Pittenger J. B. (1995). Human echolocation as a basic form of perception and action. Ecol. Psychol. 7, 181–216. doi: 10.1207/s15326969eco0703_2

[ref161] Studenski S. Perera S. Patel K. Rosano C. Faulkner K. Inzitari M.. (2011). Gait speed and survival in older adults. JAMA 305, 50–58. doi: 10.1001/jama.2010.1923 21205966 PMC3080184

[ref162] Supa M. Cotzin M. Dallenbach K. M. (1944). “Facial vision”; the perception of obstacles by the blind. Am. J. Psychol. 57, 133–183. doi: 10.2307/1416946

[ref163] Szeto B. Zanotto D. Lopez E. M. Stafford J. A. Nemer J. S. Chambers A. R.. (2021). Hearing loss is associated with increased variability in double support period in the elderly. Sensors 21:E278. doi: 10.3390/s21010278 PMC7795333 33406602

[ref164] ‘t Hart B. M. Einhäuser W. (2012). Mind the step: complementary effects of an implicit task on eye and head movements in real-life gaze allocation. Exp. Brain Res. 223, 233–249. doi: 10.1007/s00221-012-3254-x 23001370

[ref165] Teng S. Whitney D. (2011). The acuity of echolocation: spatial resolution in the sighted compared to expert performance. J. Vis. Impair. Blind. 105, 20–32. doi: 10.1177/0145482X1110500103 21611133 PMC3099177

[ref166] Thaler L. Goodale M. A. (2016). Echolocation in humans: an overview. Wiley Interdiscip. Rev. Cogn. Sci. 7, 382–393. doi: 10.1002/wcs.1408 27538733

[ref167] Thaler L. Norman L. J. De Vos H. P. J. C. Kish D. Antoniou M. Baker C. J.. (2022). Human Echolocators have better localization off Axis. Psychol. Sci. 33, 1143–1153. doi: 10.1177/09567976211068070 35699555 PMC13020946

[ref168] Thurtell M. J. Black R. A. Halmagyi G. M. Curthoys I. S. Aw S. T. (1999). Vertical eye position-dependence of the human vestibuloocular reflex during passive and active yaw head rotations. J. Neurophysiol. 81, 2415–2428. doi: 10.1152/jn.1999.81.5.2415 10322077

[ref169] Tomasi M. Pundlik S. Bowers A. R. Peli E. Luo G. (2016). Mobile gaze tracking system for outdoor walking behavioral studies. J. Vis. 16:27. doi: 10.1167/16.3.27 PMC4777240 26894511

[ref170] Tommasi D. G. Foppiani A. C. Galante D. Lovecchio N. Sforza C. (2009). Active head and cervical range of motion: effect of age in healthy females. Spine 34, 1910–1916. doi: 10.1097/BRS.0b013e3181afe826 19680100

[ref171] Valzolgher C. Todeschini M. Verdelet G. Gatel J. Salemme R. Gaveau V.. (2022). Adapting to altered auditory cues: generalization from manual reaching to head pointing. PLoS One 17:e0263509. doi: 10.1371/journal.pone.0263509 35421095 PMC9009652

[ref172] Valzolgher C. Verdelet G. Salemme R. Lombardi L. Gaveau V. Farné A.. (2020). Reaching to sounds in virtual reality: a multisensory-motor approach to promote adaptation to altered auditory cues. Neuropsychologia 149:107665. doi: 10.1016/j.neuropsychologia.2020.107665 33130161

[ref173] Van den Bogaert T. Klasen T. J. Moonen M. Van Deun L. Wouters J. (2006). Horizontal localization with bilateral hearing aids: without is better than with. J. Acoust. Soc. Am. 119, 515–526. doi: 10.1121/1.2139653 16454305

[ref174] Viljanen A. Kaprio J. Pyykkö I. Sorri M. Koskenvuo M. Rantanen T. (2009). Hearing acuity as a predictor of walking difficulties in older women. J. Am. Geriatr. Soc. 57, 2282–2286. doi: 10.1111/j.1532-5415.2009.02553.x 19874410

[ref175] Vitkovic J. Le C. Lee S.-L. Clark R. A. (2016). The contribution of hearing and hearing loss to balance control. Audiol. Neurootol. 21, 195–202. doi: 10.1159/000445100 27251708

[ref176] Vrzakova H. Bednarik R. Nakano Y. I. Nihei F. (2016). Speakers’ head and gaze dynamics weakly correlate in group conversation. Proc. Ninth Bienn. ACM Symp. Eye Track. Res. Appl., 77–84. doi: 10.1145/2857491.2857522

[ref177] Wagner P. Malisz Z. Kopp S. (2014). Gesture and speech in interaction: an overview. Speech Commun. 57, 209–232. doi: 10.1016/j.specom.2013.09.008

[ref178] Wallach H. Newman E. B. Rosenzweig M. R. (1949). The precedence effect in sound localization. Am. J. Psychol. 62, 315–336. 18134356

[ref179] Weaver T. S. Shayman C. S. Hullar T. E. (2017). The effect of hearing aids and Cochlear implants on balance during gait. Otol. Neurotol. Off. Publ. Am. Otol. Soc. Am. Neurotol. Soc. Eur. Acad. Otol. Neurotol. 38, 1327–1332. doi: 10.1097/MAO.0000000000001551 28902805

[ref180] Whitmer W. M. McShefferty D. Levy S. C. Naylor G. Edwards B. (2022). Changes in orientation behavior due to extended high-frequency (5 to 10 kHz) spatial cues. Ear Hear. 43, 545–553. doi: 10.1097/AUD.0000000000001113 34432670 PMC8862772

[ref181] Wightman F. L. Jenison R. (1995). “Auditory spatial layout” in Perception of space and motion handbook of perception and cognition. eds W. Epstein and S. Rogers. 2nd ed (San Diego, CA, US: Academic Press), 365–400. Available at: https://www.sciencedirect.com/book/9780122405303/perception-of-space-and-motion#book-description

[ref182] Wijayasinghe I. B. Ghosh B. K. (2011). Head movement dynamics constrained by Fick gimbals. IFAC Proc. 44, 9680–9685. doi: 10.3182/20110828-6-IT-1002.03777

[ref183] Wouters J. Litière L. van Wieringen A. (1999). Speech intelligibility in noisy environments with one- and two-microphone hearing aids. Audiol. Off. Organ Int. Soc. Audiol. 38, 91–98. doi: 10.3109/00206099909073008 10206518

[ref184] Yoganandan N. Pintar F. A. Zhang J. Baisden J. L. (2009). Physical properties of the human head: mass, center of gravity and moment of inertia. J. Biomech. 42, 1177–1192. doi: 10.1016/j.jbiomech.2009.03.029 19428013

[ref185] Yost W. A. Pastore M. T. Dorman M. F. (2020). Sound source localization is a multisystem process. Acoust. Sci. Technol. 41, 113–120. doi: 10.1250/ast.41.113 34305431 PMC8297655

[ref186] Zahorik P. (2002). Direct-to-reverberant energy ratio sensitivity. J. Acoust. Soc. Am. 112, 2110–2117. doi: 10.1121/1.1506692 12430822

[ref187] Zahorik P. Wightman F. L. (2001). Loudness constancy with varying sound source distance. Nat. Neurosci. 4, 78–83. doi: 10.1038/82931 11135648

[ref188] Zangemeister W. H. Jones A. Stark L. (1981). Dynamics of head movement trajectories: main sequence relationship. Exp. Neurol. 71, 76–91. doi: 10.1016/0014-4886(81)90072-8 7449898

[ref189] Zhong X. Yost W. A. (2013). Relationship between postural stability and spatial hearing. J. Am. Acad. Audiol. 24, 782–788. doi: 10.3766/jaaa.24.9.3 24224986

